# Type I Interferon Production of Plasmacytoid Dendritic Cells under Control

**DOI:** 10.3390/ijms22084190

**Published:** 2021-04-18

**Authors:** Dóra Bencze, Tünde Fekete, Kitti Pázmándi

**Affiliations:** 1 Department of Immunology, Faculty of Medicine, University of Debrecen, 1 Egyetem Square, H-4032 Debrecen, Hungary; bencze.dora@med.unideb.hu (D.B.); fekete.tunde@med.unideb.hu (T.F.); 2 Doctoral School of Molecular Cell and Immune Biology, University of Debrecen, 1 Egyetem Square, H-4032 Debrecen, Hungary

**Keywords:** plasmacytoid dendritic cells, type I interferon, regulation, antiviral response, viral infection, cancer, autoimmunity, allergy, IFN gene signature, therapy

## Abstract

One of the most powerful and multifaceted cytokines produced by immune cells are type I interferons (IFNs), the basal secretion of which contributes to the maintenance of immune homeostasis, while their activation-induced production is essential to effective immune responses. Although, each cell is capable of producing type I IFNs, plasmacytoid dendritic cells (pDCs) possess a unique ability to rapidly produce large amounts of them. Importantly, type I IFNs have a prominent role in the pathomechanism of various pDC-associated diseases. Deficiency in type I IFN production increases the risk of more severe viral infections and the development of certain allergic reactions, and supports tumor resistance; nevertheless, its overproduction promotes autoimmune reactions. Therefore, the tight regulation of type I IFN responses of pDCs is essential to maintain an adequate level of immune response without causing adverse effects. Here, our goal was to summarize those endogenous factors that can influence the type I IFN responses of pDCs, and thus might serve as possible therapeutic targets in pDC-associated diseases. Furthermore, we briefly discuss the current therapeutic approaches targeting the pDC-type I IFN axis in viral infections, cancer, autoimmunity, and allergy, together with their limitations defined by the Janus-faced nature of pDC-derived type I IFNs.

## 1. Introduction

Plasmacytoid dendritic cells (pDCs) are a specialized subset of dendritic cells (DCs), which despite their low frequency in the blood, play a crucial role in antiviral immunity and participate in the pathomechanism of several human diseases. PDCs represent a very heterogeneous and plastic cell population [1], which were initially described as a subset of cells with plasma cell-like morphology in lymph nodes in 1958, hence, the name plasmacytoid [2]. Later, in vitro studies showed that these cells share the developmental and functional features of DCs [3], and eventually were identified as professional type I interferon (IFN) producing cells (IPCs) due to their potential to produce large quantities of IFNα in response to viral stimuli [4].

Under physiological conditions, pDCs circulate in the blood or reside in secondary lymphoid organs but can hardly be found in peripheral non-immune tissues [5,6]. Nevertheless, under pathological conditions such as microbial infection, chronic inflammation, or cancer, pDCs leave the circulation and accumulate in the inflamed tissues by following the route marked by different chemotactic factors [7]. PDCs infiltrate the mucosa or skin during viral infections [8,9], and their number is also increased in tissue lesions of patients suffering from different autoimmune diseases [10]. In addition, they are present in the nasal mucosa of allergic patients, and they are also associated with different tumor tissues [10]. Under these pathological conditions, pDCs act as a double-edged sword in regulating immune responses. On the one hand, pDCs as professional IPCs are indispensable elements of antiviral immune responses, while on the other hand they can exacerbate inflammatory responses or symptoms of autoimmune diseases by the excessive production of type I IFNs, which are powerful cytokines with pleiotropic effects.

Proteins of the type I IFN family have a common helical structure composed of several long α-helices and are encoded by genes clustered on chromosome 9 in humans [11]. In humans, the multi-gene cytokine family of type I IFNs includes 13 subtypes of IFNα, only one subtype of IFNβ and single subtypes of the poorly defined IFNε, IFNκ and IFNω [12]. Human pDCs mainly express the IFNα and IFNβ subtypes, which act in an autocrine and paracrine manner to initiate cellular and intercellular processes to prevent the spread of viruses and promote the elimination of virus-infected cells [13]. Almost all cell types in the body can produce type I IFNs, mainly IFNβ, in response to viral infection, although to a much lower extent than pDCs. In addition, various microbial products and a diverse array of host factors such as cytokines and growth factors can trigger the production of type I IFNs in many cells [14].

Once secreted, type I IFNs signal through the heterodimeric transmembrane IFNα receptor (IFNAR), which is composed of the IFNAR1 and IFNAR2 subunits. The engagement of the receptor activates the tyrosine kinases Janus kinase 1 (JAK1) and tyrosine kinase 2 (TYK2), which phosphorylate the signal transducer and activator of transcription 1 (STAT1) and STAT2 transcription factors. Following that, STAT1 and STAT2 molecules dimerize and translocate to the nucleus to form the so-called IFN-stimulated gene factor 3 (ISGF3) trimolecular complex upon assembly with interferon regulatory factor (IRF) 9. ISGF3 then binds to IFN-stimulated response elements (ISREs) and results in the transcription of several hundreds of IFN-stimulated genes (ISGs). ISG-encoded proteins induce the establishment of an antiviral state in infected and neighboring cells to prevent viral replication and the dissemination of the pathogen, thus type I IFNs are a powerful tool to tackle viral infections [14,15]. Among the IFN-induced proteins, many enzymes such as the RNA-dependent protein kinase (PKR), the 2′,5′-oligoadenylate (Oligo A) synthetase (OAS), the ribonuclease L (RNase L) and the myxovirus resistance guanosine triphosphatases (Mx GTPases) are upregulated and implicated in the protection against viral infection. In particular, PKR changes the translational pattern of the host cell by phosphorylating the α subunit of eukaryotic initiation factor 2 (eIF2α), which leads to the transient suppression of protein synthesis and the consequent prevention of viral replication [16]. Upon binding to dsRNA, OAS starts to synthesize Oligo A, which as a second messenger activates RNase L to degrade viral RNA [17]. Moreover, Mx protein GTPases self-assemble into oligomers and block the intracellular transport of viral nucleocapsid or nucleocapsid-like structures, thus these viral components become trapped and unavailable for the generation of new virus particles [18].

In addition to eliciting an antiviral state, type I IFNs as potent pleiotropic cytokines fine-tune innate immune responses by promoting antigen presentation, supporting natural killer (NK) cell functions while limiting the excess production of inflammatory cytokines [14]. In particular, IFNα promotes the recruitment of monocytes into the inflamed tissues and their differentiation into effective antigen presenting cells (APCs) [19,20]. Moreover, type I IFNs induce DC maturation and activation [21]. At later stages of infection, type I IFNs activate adaptive T and B cell responses and promote the development of immunological memory. Via supporting the production of B lymphocyte stimulators such as B cell activating factor (BAFF) and A proliferation-inducing ligand (APRIL) by DCs and macrophages, type I IFNs have also been reported to improve B cell survival, maturation, differentiation, and class-switch recombination [22]. Furthermore, upon acute infections, type I IFNs support the activation and expansion of antigen-specific CD4+ helper T (Th) cells and CD8+ cytotoxic T cells, and contribute to the differentiation of follicular Th cells, which are critical to the induction of B cell responses [23,24].

**Figure 1:**
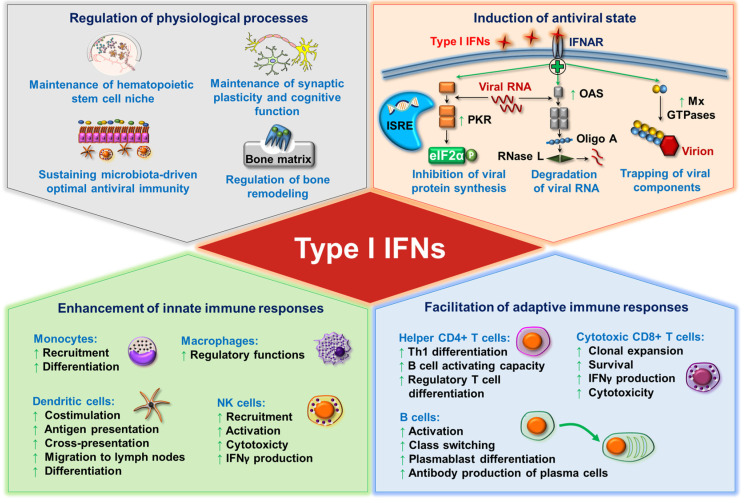
**The pleiotropic effects of type I interferons (IFNs).** Continuous baseline production of type I IFNs by various tissues and cells fine-tunes a wide variety of physiological processes including hematopoietic stem cell functions, synaptic plasticity, bone remodeling and immune homeostasis. In addition, the microbiota-induced basal IFN-signature prepares stromal and immune cells for upcoming infections (**upper left panel**). Upon viral infection, type I IFN signaling induces antiviral state in all nucleated cells via the upregulation of IFN-stimulated genes that inhibit the replication and spreading of viruses (**upper right panel**). Type I IFNs also control the cells of innate (**lower left panel**) as well as adaptive (**lower right panel**) immune system by shaping the activation, differentiation, effector functions and trafficking of these cells. *eIF2α: eukaryotic initiation factor 2α; IFN: interferon; IFNAR: interferon-alpha/beta receptor; ISRE: IFN-stimulated response element; Mx GTPase: myxovirus resistance guanosine triphosphatase; NK: natural killer; OAS: 2′-5′ oligoadenylate synthetase; Oligo A: 2′-5′-oligoadenylate; PKR: protein kinase R; Rnase L: ribonuclease L; Th: T helper.*

Besides regulating innate and adaptive immune cell activation, survival, and differentiation, type I IFNs also contribute to the control of various physiological processes such as the regulation of hematopoietic stem cell niche function, bone remodeling, the maintenance of synaptic plasticity and cognitive function of the healthy central nervous system, and the maintenance of immune homeostasis [25,26]. Furthermore, commensal microbiota-driven tonic levels of IFN signals at mucosal surfaces adjust the activation threshold of the innate immune system, which is essential for ideal antiviral responses [27,28,29] (Figure 1).

Under physiological conditions, in the absence of acute infection, type I IFNs are constitutively secreted at a baseline level in many tissues, and it appears that lower or higher amounts than that might have pathologic consequences [25]. Despite their beneficial effects, type I IFNs are detrimental to the host when their expression is dysregulated. While the acute and transient production of type I IFNs promote antiviral responses, their sustained and chronic secretion drives various autoimmune and non-autoimmune inflammatory diseases. These diseases are characterized by the so-called IFN gene signature (IGS), which refers to the upregulation of IFN inducible genes in peripheral blood cells. IGS was found to be correlated with disease severity in patients with autoimmune diseases such as systemic lupus erythematosus (SLE), rheumatoid arthritis (RA) or dermatomyositis [30].

Owing to their potential to secrete large quantities of type I IFNs, pDCs have been identified as major players in a number of type I IFN-mediated inflammatory conditions. Since the discovery of pDCs, several regulatory factors and mechanisms have been identified, which affect their type I IFN production and might serve as possible therapeutic targets. In the present review, first we briefly introduce the molecular basis of the unique type I IFN producing capacity of pDCs. Following that, we summarize all those endogenous factors, which can modulate type I IFN production specifically in pDCs and outline the role of pDC-derived type I IFNs in antiviral response, cancer, autoimmunity, and allergy, and shortly discuss the potential therapeutic approaches in these diseases.

## 2. Professionalism of pDCs in the Production of Type I IFNs

Despite being discovered in the mid-20th century, pDCs were explicitly characterized many decades later due to their low frequencies in peripheral blood and rapid apoptosis under in vitro culture conditions. After the ability of these cells to secrete extreme amounts of type I IFNs in a relatively short time was recognized, they were defined as natural IPCs. According to the current model, IPCs are precursors of pDCs, and therefore the IFN-producing form of pDCs is regarded as plasmacytoid pre-DCs. Plasmacytoid pre-DCs are small, round cells with a plasma cell-like morphology and a well-developed endoplasmic reticulum (ER), which enables the production of large quantities of proteins. Upon differentiation into mature pDCs, they lose their exceptional type I IFN-producing ability, take up DC-like morphology, express high levels of major histocompatibility complex (MHC) and costimulatory molecules, and become professional APCs being capable of stimulating naive T cells [31].

Plasmacytoid DCs have emerged as “the virus experts” of our body owing to their striking capacity to produce large amounts of type I IFNs. Within 6 h of viral exposure, pDCs devote 50% of their induced transcriptional activity to initiate type I IFN gene expression [32]. Human pDCs express a wide repertoire of type I IFNs including 13 subtypes of IFNα and single subtypes of IFNβ, IFNω, and IFNτ [33]. In response to viral infection, pDCs are responsible for 95% of type I IFN production by mononuclear cells, as they are able to produce 200–1000 times more type I IFNs than any other white blood cell after microbial exposure [4]. Quantitatively, one single pDC can produce 3–10 pg of IFNα in response to a strong stimulus. While secreting high amount of IFNα, pDCs produce much lower levels of IFNβ and additional type I IFNs [34]. All these data raise the question of how pDCs are capable of such a peak performance. In contrast to conventional DCs (cDC), pDCs selectively express endosomal Toll-like receptor (TLR) 7 and 9, which recognize viral RNA and DNA, respectively, and their activation is associated with a high production of type I IFNs by pDCs [35]. In contrast to other cell types, pDCs show a high degree of resistance to viral infections, and do not need to be infected with live viruses to induce the production of type I IFNs [36], since inactivated viruses can also stimulate pDCs if the viral envelope remains intact [37]. The internalized virus particles are degraded within endocytic vesicles and are sensed through TLR7 and TLR9, the engagement of which induces signaling through the adaptor protein MyD88. Upon association with downstream signaling components, MyD88 leads to the phosphorylation and nuclear translocation of the IRF7 transcription factor, which initiates the transcriptional activation of type I IFN genes. In contrast to cDC, in which IRF7 is inducible and requires prior stimulation, pDCs constitutively express IRF7, possibly due to the lack of the translational repressor eukaryotic translation initiation factor 4E (eIF4E)-binding protein (4E-BP), which allows the rapid onset of type I IFN production in pDCs [38]. However, constitutive IRF7 expression alone would not be sufficient to induce the production of large amounts of type I IFNs in pDCs. Honda et al. described a unique spatiotemporal regulation of the TLR9-MyD88 pathway in pDCs in response to a specific TLR9 ligand, CpG-A. Following recognition, CpG-A oligonucleotides form large multimeric aggregates, which are retained in the early endosomes of pDCs for about 30 min that allows prolonged IRF7 induction, whereas in cDCs, those are rapidly transferred to lysosomal compartments. Thus, CpG-A stimulation efficiently enhances the production of type I IFNs in pDCs via IRF7, whereas barely affects nuclear factor-κ B (NF-κB) activity and thus the maturation of these cells. However, it is important to note that this effect highly depends on the structural properties of the TLR ligand. Other subtypes of oligonucleotides such as the monomeric CpG-B is rapidly transported to late endosomes, where recognition through TLR9 leads to the activation of the NF-κB pathway, which induces the synthesis of inflammatory cytokines, chemokines, and costimulatory molecules; therefore, CpG-B is much less effective in the initiation of type I IFN production compared to CpG-A [39]. In terms of kinetic, pDCs secrete high amounts of IFNα within the first 12 h of exposure to CpG-A or live viruses, and then in the next 48 h, pDCs are able to produce only a small fraction of this quantity upon re-stimulation [33].

Previously, our research group proposed a model, in which type I IFN secretion by pDCs occurs in two waves under the coordinated regulation by different subtypes of pattern recognition receptors (PRRs) [40]. In the early phase of viral infection, pDCs are able to detect debris from virus-infected cells in the lymph nodes through constantly expressing TLR7 and TLR9 receptors that results in the secretion of high levels of type I IFNs and subsequent induction of a systemic antiviral state. The systemic effect is facilitated by the unique localization of pDCs, in which aspect they highly differ from cDCs that are generally located in peripheral tissues, i.e., at the sites of viral entry, whereas pDCs are found in the blood or in lymphoid tissues such as lymph nodes. Therefore, pDCs are initially not infected, but can detect virus-infected cell debris delivered to lymph nodes and produce a large amount of antiviral cytokines, which might reach every cell in the body through the blood or lymphoid circulation, and thus contributes to the development of a systemic antiviral state. In the later stage of viral infection, TLR-activated pDCs migrate to the site of virus entry, where due to the high viral load, they can also get infected. However, it is important to note that previous TLR stimulation induces the expression of cytosolic retinoic acid-inducible gene I (RIG-I)-like receptors (RLRs) in pDCs, the expression of which is only marginal in these cells without prior activation. Therefore, pDCs gain the ability to recognize replicating viruses even in the cytoplasm after TLR stimulation. Upon ligand engagement, RLRs recruit the mitochondrial antiviral signaling adapter protein (MAVS), which leads to the activation of MAVS-dependent IRF3/7 pathways and ultimately results in a second/late wave of type I IFN production. The RLR-mediated type I IFN production is lower in amounts than the TLR-induced secretion by pDCs; however, it still effectively supports potent local antiviral responses [40].

Based on single-cell genomic profiling several models have recently been proposed on the fate and functional plasticity of pDC [1,36,41]. According to these models, pDCs highly differ in their degree of differentiation and consequently in their capacity to produce type I IFNs or present antigens. A current study demonstrated that the same individual pDC first produces type I IFNs and then acquires the ability to present antigens to T cells during in vivo viral infections suggesting that pDCs exert different functions orchestrated in a spatiotemporal manner [42]. Other models suggest that only a small fraction of pDCs can produce type I IFNs, regardless of the stimulus. It is also plausible that while some pDCs get infected, others recognize the infected pDCs and start to produce the type I IFNs as a response [1]. The above data indicate that further studies are required to explain the observed discrepancies and clarify whether the functional differences of pDCs are due to terminal functional specialization or are subsequent stages of pDC maturation.

In conclusion, pDCs represent the number one source of type I IFNs, which cytokines impact an array of cellular events, physiological processes and both innate and adaptive immune responses. Therefore, fine-tuning type I IFN responses of pDCs by different endogenous factors or regulatory mechanisms is essential to keep a balance between protection and unwanted pathological events.

## 3. Regulation of Type I IFN Production at the Transcriptional and Posttranscriptional Level

### 3.1. Transcription Factors

The induction of type I IFN secretion is primarily controlled at the transcriptional level, where IRFs play an essential role. In pDCs, IRF7 and IRF3 are the master regulators of type I IFN responses [40,43]. As we previously described, the MyD88-dependent TLR7/9 signaling cascade activates IRF7 to induce robust production of type I IFNs, whereas the MAVS-dependent RLR signaling pathway is able to activate both IRF3 and IRF7 to promote the secretion of type I IFNs in pDCs [40,44]. In addition, other members of the IRF family have been implicated in the regulation of type I IFN production [45].

Several studies demonstrated that IRF5 and IRF8 also contribute to the regulation of type I IFN production. In mouse pDCs, IRF5 was found to only partially affect the induction of IFNα, whereas it seems to be critical for IFNβ gene induction [46,47]. In the human CAL-1 pDC cell line, IRF5 silencing resulted in an 80% reduction in CpG-B-triggered gene activation as compared with controls [48]. Interestingly, the same study identified IRF8 as a negative regulator, since silencing of IRF8 led to a 60% increase in gene activation upon CpG stimulation [48]. Moreover, the authors also found that IRF5 and IFR8, which colocalize within the cytoplasm of resting pDCs, rapidly translocate to the nucleus after CpG triggering, and thus hypothesize that IRF8 interacts with IRF5 to control TLR9 signaling in human pDCs [48]. By contrast, another study demonstrated that the depletion of IRF8 decreased the IFNα secreting capacity of mouse pDCs upon CpG stimulation indicating its importance in mouse pDCs [49]. Thus, the above data suggest that IRF8 controls the magnitude of IFN responses and exerts positive or negative regulatory effects depending on the origin of the cell.

Besides IRFs, several other transcription factors were identified as regulators of pDC functions. Runt-related transcription factor 2 (RUNX2) is essential for the optimal production of type I IFNs through the modulation of IRF7 expression in mice. In the absence of RUNX, both resting and CpG-activated mouse bone marrow (BM)-derived pDCs showed decreased IRF7 expression, which resulted in a significantly reduced production of IFNα and IFNβ [50]. Further, mouse pDCs highly express the Ets family transcription factor, Spi-B, which can transactivate the promoters of type I IFN in synergy with IRF7 [51]. BM-derived and splenic pDCs from Spi-B-deficient mice showed defective induction of IFNα genes following CpG-B, polyuridylic acid (polyU) and vesicular stomatitis virus (VSV) stimulation. The authors also concluded that constitutive high expression of Spi-B contributes to the ability of pDCs to produce high amounts of type I IFNs in response to TLR7 and TLR9 ligands [51]. The nuclear factor of activated T cells C 3 (NFATC3) was also found to enhance IRF7-mediated IFN release by both mouse and human pDCs [52]. In BM-derived pDCs from NFATC3-deficient mice TLR7/9-mediated IFNα production was greatly reduced compared to wild type mice [52]. In the Gen2.2 human pDC cell line, the knockdown of NFATC3 also led to a substantial reduction of IFNα production in response to CpG-A. Furthermore, it was demonstrated that NFATC3 forms a complex with IRF7 and binds to IFN promoters to augment maximal production of type I IFNs upon TLR9 stimulation in GEN2.2 cells [52].

In contrast to the above mentioned transcription factors, the pleiotropic transcription factor MYC negatively regulates the TLR-mediated antiviral response of human pDCs [53]. The knockdown of MYC increased the CpG-B triggered induction of IFN-stimulated genes in the human GEN2.2 pDC cell line. In particular, MYC is shown to interact and form a complex with the nuclear receptor co-repressor 2 (NCOR2) and histone deacetylase 3 (HDAC3) to occupy, and thus repress the promoter region of IRF7. These data imply that MYC suppresses IRF7 promoter activity to ensure the optimal levels of type I IFN production and prevent the development of autoimmune diseases [53].

Another critical regulator of various signaling pathways, the CXXC type zink finger protein 5 (CXXC5) is suggested to act as a transcription factor as well as an epigenetic modifier [54]. It is highly expressed in mouse and human pDCs, where, as an epigenetic regulator it controls DNA methylation and histone modifications [55]. Following stimulation with herpes simplex virus-1 (HSV-1) or CpG-A, pDCs from CXXC5-deficient mice expressed lower levels of IRF7 and produced much less type I IFN compared to control pDCs. Mechanistically, CXXC5 recruits the Tet2 DNA demethylase, which maintains hypomethylation of CpG-island containing genes such as IRF7, and thus contributes to the rapid and robust type I IFN production of mouse pDCs [55]. Similarly, the knockdown of CXXC5 in the human GEN2.2 pDC cell line led to reduced IRF7 expression and decreased mRNA levels of IFNα and IFNβ upon exposure to the TLR7 ligand R848 and HSV-1 [55]. Furthermore, E2-2 is also a specific regulator of mouse as well as human pDCs, since it can directly activate the expression of multiple genes involved in pDC-mediated type I IFN responses, namely the TLR7 and TLR9 receptors as well as the IRF7, IRF8 and Spi-B transcription factors [56]. The deletion of E2-2 in mouse BM-derived pDCs abolished type I IFN release in response to CpG-A that could be explained by the reduced expression of the aforementioned TLR pathway components [56]. Similarly, E2-2 knockdown in the human GEN2.2 cell line downregulated the expression of pDC signature genes and diminished IFNα production in response to CpG-B. Interestingly, E2-2 silencing in GEN2.2 cells upregulated a set of cDC specific genes, including the anti-inflammatory TLR10 and inhibitory Siglec-6 receptors that could explain the abrogated IFNα production in response to TLR9 stimulation [57].

The above data indicate that several transcription factors act in concert to coordinate the optimal expression of type I IFNs in both human and mouse pDCs (Table 1).

### 3.2. Adaptor Proteins and Other Intracellular Regulators

Besides the common downstream signaling components of TLR and RLR pathways, numerous adaptor proteins and intracellular molecules act as positive or negative regulators of type I IFN secretion by pDCs.

Among them, the glycoprotein osteopontin (Opn) seems to be essential to the type I IFN responses in pDCs. Many cell types are able to express Opn, which is involved in various pathophysiological events when secreted [73]. However, novel studies found that a portion of newly synthesized Opn is retained in the cytoplasm (referred as Opn-i), where acting as an adaptor protein controls signal transduction pathways downstream of innate immune receptors [73]. In splenic mouse pDCs, Opn deficiency significantly reduced the production of IFNα in response to CpG-A or CpG-B stimulation [58]. Opn-i colocalizes with MyD88 and TLR9 that is essential to the nuclear translocation of IRF7, and thus the optimal induction of IFNα gene expression [58]. Furthermore, the cytoplasmic phosphoproteins Protein kinase C and casein kinase substrate in neurons 1 (PACSIN1) was also identified as a pDC-specific adaptor molecule critical for the TLR7/9-mediated type I IFN responses in both human and mouse pDCs [59]. The knockdown of PACSIN1 in human GEN2.2 cells inhibited IFNα responses to CpG-A stimulation. In addition, the depletion of PACSIN1 in mouse BM-derived pDCs considerably reduced the levels of IFNα production in response to CpG-A, influenza virus (Flu), VSV and HSV-1. Since Flu and VSV are recognized via TLR7, whereas HSV-1 and CpG are sensed by TLR9, the authors concluded that PACSIN1 affects IFNα production by both TLR7 and TLR9 signaling pathways in pDCs [59].

Several members of the tripartite motif (TRIM)-containing proteins have also emerged as important modulators of innate immune signaling cascades including the type I IFN pathway of pDCs. In primary human pDCs, TRIM20, TRIM22, TRIM28, and TRIM36 were identified as negative regulators of type I IFN responses [60]. On the contrary, TRIM8, which is constitutively expressed in resting pDCs, was revealed as a strong positive regulator of type I responses of pDCs and therefore its regulatory mechanisms were further investigated [60]. The silencing of TRIM8 in pDCs led to a profound decrease in IRF7 phosphorylation and abolished type I IFN production in response to HIV or Flu virus infection. The authors also showed with mechanistic studies on HEK293T cells that TRIM8 prevents the proteasomal degradation of phosphorylated IRF7 through inhibiting its recognition by the peptidyl-prolyl isomerase Pin1 [60].

Another positive regulator is the phospholipid scramblase 1 (PLSCR1) protein, which interacts directly with several plasma membrane receptors, and acting as a scramblase, is involved in multiple biological processes [74]. Silencing of PLSCR1 in human GEN2.2 cells led to a significant reduction of IFNα responses following CpG-A or CpG-B stimulation [61]. Similar results were also obtained with BM-derived pDCs from PLSCR1-deficient mice when stimulated with CpG-A, Flu virus and HSV-1. In one human pDC cell line, PLSCR1 plays an important role in TLR9 trafficking from the ER to the early endosomes, and thus supports its engagement with synthetic or viral ligand [61].

Recently, it was reported that the lipid converting enzyme Sphingosine kinase 1 (SphK1), which catalyzes the formation of the lipid signaling molecule sphingosine-1-phosphate (S1P), plays a critical role in pDC functions as well [62]. The depletion of ShpK1 in mouse pDCs or pre-treatment of human CAL-1 cells and mouse splenic pDCs with ShpK1-specific inhibitors significantly impaired TLR7/9-mediated type I IFN production [62]. Mechanistically, ShpK1 was found to regulate the nuclear transport of IRF7 as well as the uptake and trafficking of CpG to endosomes, where TLR9 activation occurs. Further, pharmacological inhibition of SphK1 or its genetic deletion in a mouse model of SLE decreased pDC activation and ISGs expression [62].

In addition, the lysosomal membrane protein Scavenger receptor class B member 2 (SCARB2) is highly expressed in resting pDCs, and its expression can be further upregulated by CpG stimulation [63]. Interestingly, the silencing of SCARB2 in human GEN2.2 cells significantly reduced the CpG-B-mediated IFNα production, whereas did not affect it upon CpG-A triggering. Further studies revealed that SCARB2 localizes in the late endosome, where it mediates the endosomal transport of TLR9 as well as nuclear translocation of IRF7, and thus regulates the expression of IFNα in pDCs [63].

Besides the aforementioned regulatory factors, the major drivers of immunometabolic changes can also impact type I IFN production of pDCs. Immunometabolism is one of the hottest topics and a dynamically growing field of immunology, which can provide new therapeutic approaches for the treatment of immune-related diseases. Immune cell functions can be shaped both by intracellular metabolic processes and external metabolites derived from pathogens, microbes, or tumor cells [75]. Most importantly, several papers reported that alterations in energy and lipid metabolism modulate the type I IFN producing ability of pDCs [76,77,78]. The mammalian target of rapamycin (mTOR) has emerged as the master regulator of cellular metabolism by controlling a myriad of cellular functions in response to environmental factors and intracellular signals. It was revealed first in 2008 that the phosphatidylinositol 3-kinase (PI3K)-Akt-mTOR signaling pathway is crucial for the TLR-mediated type I IFN responses of pDCs [64]. The inhibition of mTOR complex 1 by rapamycin significantly decreased the TLR9-triggered production of IFNα both in mouse and human pDCs. Moreover, the inhibition of the PI3K upstream molecule or p70S6 kinase downstream target also substantially suppressed the CpG-A-induced IFNα secretion by mouse pDC. Furthermore, the TLR7-induced IFNα secretion was also impaired in murine splenic pDCs. The findings also indicated that blockade of mTOR signaling results in the disruption of MyD88-TLR9 complex and impairment of IRF7 phosphorylation and nuclear translocation [64]. Another group further confirmed these data by showing that suppressing the signaling components of the PI3K-Akt-mTOR pathway by various approaches significantly reduced IFNα secretion in primary human pDCs [79]. A different study also demonstrated that rapamycin potently inhibits TLR7/9-induced IFNα secretion in peripheral blood pDCs [80]. In addition, our research group demonstrated for the first time that mTOR signaling is also essential to the RLR-triggered antiviral immune responses of pDCs. We described that mTOR blockade by rapamycin or the dual kinase inhibitor AZD8055 inhibited the type I IFN production in primary human pDCs and GEN2.2 cells upon RLR stimulation [65]. Further, we found that mTOR blockade decreased the RLR-triggered phosphorylation of Tank-binding kinase 1 (TBK1), which based on literature data is a requirement for IRF3/7 activation [81]. Thus, we hypothesize that mTOR might support the RLR-initiated type I IFN production of pDCs via regulating at the level or upstream of TBK1.

It has long been recognized that several metabolic processes are linked to the enhanced generation of mitochondrial reactive oxygen species (mtROS), which are also essential mediators of immune responses. MtROS are constitutively generated during physiological conditions and their production can be further increased under pathological states. As important signaling molecules, mtROS participate in various processes of immune cell functions such as activation or inflammatory cytokine production [82]. Interestingly, BM-derived pDCs of aged mice show defective IRF7 upregulation upon TLR9 activation as compared with cells from younger counterparts [83]. Both resting and TLR9 activated aged pDCs displayed elevated levels of reactive oxygen species (ROS), the reduction of which by antioxidant pre-treatment restored IFNα production during TLR9 activation. These results suggest that age-induced oxidative stress impairs the antiviral capacity of pDCs in response to TLR9 stimulation, which might explain the increased susceptibility of older individuals to viral infections. In addition, our research group described that mtROS can influence the type I IFN producing capacity of human pDCs [66]. The TLR9 agonist CpG-A triggered the type I IFN production of human GEN2.2 cells was markedly reduced by high mtROS levels, which inhibited the phosphorylation of IRF7. On the contrary, elevated mtROS increased RIG-I-stimulated type I IFN expression and augmented the phosphorylation of Akt and IRF3, which are essential components of RLR signaling [66]. Interestingly, in a separate study, we demonstrated that exogenous ROS also have a negative effect on the TLR7-dependent activation of pDCs [67]. Thus, our data suggest that the effects of mtROS on pDCs depend on which viral sensing pathways are stimulated. Namely, the early TLR7/9-mediated type I IFN response is abrogated, whereas the late RLR-mediated production of type I IFNs is supported by elevated mtROS levels. (Table 1)

Although, the above listed regulatory molecules have multifunctional roles in the regulation of various physiological cellular functions, those are also important in fine-tuning the extent of type I IFN responses by pDCs as well. It is also noteworthy that through feedback mechanisms secreted type I IFNs might also act on diverse cellular functions including metabolic processes, which highly influence the activation of immune cells.

### 3.3. MicroRNAs

MicroRNAs (miRs) are short, non-coding RNAs, which upon binding to target mRNAs lead to their degradation or translational suppression. By controlling gene expression at the posttranscriptional level in various cell types, miRs have emerged as key coordinators of both innate and adaptive immune responses [84], and several of them have also been linked to the regulation of type I IFN responses in pDCs [85].

MiRs profiling of human pDCs revealed that TLR7 stimulation highly induced the expression of both the guide (miR) and passenger strands (miR*) of 19 different miRNAs, among which miR-155 and miR-155* were the most highly induced ones. Interestingly, miR-155 inhibited the TLR7-triggered production of IFNα and IFNβ expression by targeting TGFβ activated kinase 1 binding Protein 2 (TAB2), whereas miR-155* augmented it by suppressing interleukin-1 receptor-associated kinase (IRAK)-M [68].

Similarly, miR-146a expression was also induced upon TLR7/9 stimulation in primary human pDCs [69]. However, the silencing of miR-146a increased the percentage of IFNα expressing pDCs upon stimulation with CpG-A, thus it was identified as a negative regulator of type I IFN production [69]. Further studies with the CAL-1 pDC cell line also revealed that miR-146a targets IRAK-1, which is an essential element for IRF7 activation [69].

Interestingly, increasing evidence has revealed that the dysregulation of miR expression can contribute to the development and maintenance of various autoimmune diseases [86]. So far, two studies revealed association between miR expression and type I IFN production of pDCs in patients with IFN signature. One study found that pDCs of systemic sclerosis patients upregulated miR-618, the overexpression of which in primary human pDCs resulted in a higher secretion of IFNα in response to the TLR9 stimulation compared to pDCs derived from healthy individuals [70]. Another study reported that pDCs of SLE and antiphospholipid syndrome (APS) patients with high IFN signature displayed reduced expression of miR-361-5p, miR-128-3p and miR-181a-2-3 p compared to pDCs from patients without an IFN signature or healthy controls [87]. Circulating pDCs from Sjögren’s patients with an IFN signature also showed decreased expression of several miRs, however, no strong correlation was found between the miR profile of pDCs and IFN signature of patients [88].

The role of miRs in the regulation of type I IFN responses by pDCs was also proven in mouse models. In mouse BM-derived pDCs, both TLR7 and TLR9 stimulation increased the expression of different miRs such as miR-21, which was found to be an essential positive regulator for IFNα. The phosphatase and tensin homolog (PTEN) was identified as the target of miR-21. By suppressing PTEN, miR-21 promotes the TLR7/9-mediated activation of PI3K-Akt-mTOR signaling, which is essential for IRF7 protein expression and subsequent IFNα production [71]. Furthermore, miR-126, which is mostly associated with the regulation of vasculogenesis and angiogenesis, is also able to control IFNα and IFNβ production of mouse pDCs. MiR-126 deficient mice produced less IFNα than wild-type mice in response to TLR7 (R848) and TLR9 (CpG-A) agonists [72]. Splenic and lymph node-derived pDCs from miR-126-/- mice were also defective in their TLR9-mediated IFNα producing capacity compared to wild type pDCs [72]. Further data indicate that tuberous sclerosis complex 1 (TSC1), which is a negative regulator of mTOR, is the target for miR-126 in pDCs [72]. It was also hypothesized that miR-126 acts through the upregulation of vascular endothelial growth factor receptor 2 (VEGFR2), which also negatively regulates TSC1, thus increases mTOR activity and type I IFN production in pDCs [72] (Table 1).

Altogether, miRs can impact the type I IFN release of pDCs both in a positive or negative manner and might represent potential biomarkers for the diagnosis and prognosis of certain type I IFN-mediated autoimmune diseases.

## 4. Regulation of Type I IFN Production by Receptor Interactions

### 4.1. Activating Receptors of Type I IFN Production

Receptor-mediated regulatory mechanisms influence various events in a cell’s life. PDCs express a wide range of cell-surface and intracellular receptors, which upon interaction, might affect the outcome of pDC activation [1]. Interestingly, only a few of these immunoregulatory receptors have the ability to support pDC functions, and more specifically their type I IFN producing capacity.

The first identified positive regulator of type I IFN responses of pDCs is represented by the immunoregulatory CD300a/c molecule. It was found that the cross-linking of CD300a/c increased IRF7 expression and IFNα secretion by primary human pDCs after TLR7/9 activation [89]. Later, it was reported that pDC specific triggering receptor expressed on myeloid cells (PDC-TREM), which expression requires TLR7/9 stimulation, also positively affects the type I IFN production of pDCs. Upon stimulation of mouse BM-derived pDCs with CpG-A, PDC-TREM forms a complex with endogenous Plexin-A1 and its endogenous ligand Sema6D, which leads to the phosphorylation of PI3K, extracellular-signal-regulated kinase 1/2 (ERK1/2) and inhibitory kappa B kinase α (IKKα), and eventually results in a robust production of type I IFNs by pDCs [90].

Interestingly, despite the presence of the inhibitory cytoplasmic immunoreceptor tyrosine-based inhibition motif (ITIM), Ly49Q is a positive regulator of type I IFN responses in mouse pDCs as well. In mice, the interaction of Ly49Q receptor with its ligand, the classical MHC-I molecule, was found to be required for IFNα secretion by pDCs, since splenic pDCs from Ly49Q-deficient mice displayed lower levels of IFNα upon CpG-A challenge [91]. Moreover, Ly49Q-/- mice were defective in systemic IFNα production. Blockade of either Ly49Q or its ligand by monoclonal antibodies (mAB) almost completely abrogated the IFNα secretion by pDCs. These findings suggest that Ly49Q and MHC-I linkage positively regulates TLR9 mediated type I IFN production in mice [91]. As a mechanism, the research group demonstrated that Ly49Q controls the intracellular trafficking of TLR9/CpG-A containing vesicular compartments [92]. These data were supported by another study showing that Ly49Q deficient mouse BM-derived pDCs produced lower amounts of IFNα and IFNβ relative to control pDCs in response to CpG-B and Sendai virus [93]. As another mechanism, later it was revealed that Ly49Q enhances TLR9-mediated IRF7 nuclear translocation, and thus type I IFN gene expression in an ITIM-dependent manner in mouse pDCs as well [94].

In addition, the signaling lymphocyte activation molecule family 9 (SLAMF9) receptor is highly expressed on pDCs and plays and important role in their differentiation and function. In mice, SLAMF9 deficiency results in the accumulation of pDCs in the lymph nodes, where they exhibit reduced costimulatory potential, and show a decreased capacity to secrete IFNα in steady-state condition and during experimental autoimmune encephalomyelitis (EAE). Moreover, SLAMF9-/- pDCs derived from EAE mice and CpG-A stimulated mice show a reduction in IFNα levels compared with the wild type controls suggesting that SLAMF9 might impact pDC functionality under different inflammatory conditions. A gene expression analysis of SLAMF9 deficient pDCs further revealed that the levels of the SpiB transcription factor was strongly downregulated that might contribute to the impaired functionality of pDCs in the periphery [95].

Furthermore, pDCs express the multifunctional receptor for advanced glycation endproducts (RAGE), which is able to bind multiple ligands via recognizing a common structural motif in them, and thus it is regarded as a PRR [96]. An important ligand for RAGE is the nuclear protein high mobility group box 1 protein (HMGB1), which when released by dying cells acts as a proinflammatory factor. In 2005, it was demonstrated that purified human pDCs express RAGE, and upon TLR9 stimulation secrete HMGB1, which, acting in an autocrine manner, supports the maturation and type I IFN secretion of pDCs [97]. Another study demonstrated that HMGB1 released by pDCs and NK cells triggers IFNα secretion of pDC in the context of HIV infection in humans [98]. By supporting IFN secretion, HMGB1 contributes to the upregulation of tumor necrosis factor-related apoptosis-inducing ligand (TRAIL) on pDCs, which makes them able to kill death receptor 5 (DR5) expressing CD4+ T cells. Moreover, HMGB1 released from necrotic cells can bind to DNA containing immune complexes, which then positively regulate IFNα secretion by pDCs. HMGB1 in complex with CpG-A oligonucleotides significantly increased the IFNα production in mouse BM-derived pDCs compared to CpG-A alone through inducing the association of RAGE with MyD88-TLR9 [99]. In obese individuals, by transporting extracellular DNA through RAGE to TLR9, adipose tissue derived HMGB1 triggers pDCs to produce IFNα, which then drives the proinflammatory polarization of resident macrophages and contributes to the development of systemic insulin resistance [100] (Table 2).

### 4.2. Inhibitory Receptors of Type I IFN Production

Many studies have reported that the cross-linking of regulatory cell surface receptors on pDCs efficiently suppresses their ability to produce type I IFNs. Several of these regulatory receptors associate with immunoreceptor tyrosine-based activation motif (ITAM) containing adapter proteins such as DNAX activation protein 12 (DAP12) and Fc receptor (FcR) γ-chain (FcRγ) or contain ITIM motifs themselves, which mediate inhibitory signals [129]. FcRγ is often referred to as FcεRIγ since it was first discovered as the third subunit of FcεRI [130]. Later, it was revealed that it is a common subunit of various FcRs and is also able to associate with a number of immune receptors such as blood dendritic cell antigen 2 (BDCA2) and immunoglobulin-like transcript 7 (ILT7) [131].

BDCA2 (also known as CD303) is a type II C-type lectin receptor, which is exclusively expressed by human pDCs. In 2001, it was demonstrated that the ligation of BDCA2 with a specific antibody suppresses the ability of peripheral blood-derived human pDCs to produce IFNα in response to CpG-A [101]. Later, co-immunoprecipitation experiments revealed that BDCA2 forms a complex with the transmembrane adapter FcRγ, which interferes with the TLR7/9-mediated type I IFN producing ability of purified human pDCs [102]. A separate study with primary human pDCs revealed further details of BDCA2 and FcRγ interaction by showing that upon association with FcRγ BDCA2 signals through a B cell receptor (BCR) signalosome like complex consisting of Lyn, spleen tyrosine kinase (Syk), Bruton tyrosine kinase (Btk), Src homology 2 (SH-2) domain-containing leukocyte protein of 65 kDa (Slp65) and phospholipase C-gamma 2 (PLCγ2) [103]. Moreover, a subsequent study with GEN2.2 cells and primary human pDCs declared that the BCR-like signaling activates the mitogen-activated protein kinase (MAPK) kinase (MEK)1/2-ERK pathway, which then upregulates c-Fos, and thus results in the inhibition of CpG-A mediated type I IFN production [132]. Further data indicate that the CD2-associated adaptor protein (CD2AP), which is specifically expressed by pDCs, forms a complex with SH-2 domain-containing inositol-5-phosphatase 1 (SHIP1) and inhibits the Casitas B cell lymphoma (Cbl)-mediated ubiquitination and degradation of FcRγ [104]. Thus, via supporting BDCA2/FcRγ receptor signaling, CD2AP negatively controls TLR9-induced type I IFN responses in pDCs [104]. Interestingly, some studies identified a few possible virus-derived ligands for BDCA2, in particular, hepatitis B virus surface antigen (HBsAg) and HIV-1 glycoprotein 120 (gp120). The binding of HBsAg [133] or gp-120 [134] to BDCA2 on the surface of human pDCs contributed to the suppression of IFNα secretion in response to TLR9.

The above data indicate that targeting BDCA2 by blocking or depleting antibodies might be a promising approach to manage type I IFN-mediated pathologies including autoimmune diseases [135,136,137]. Importantly, certain mAbs are already under clinical testing for the treatment of SLE and cutaneous lupus erythematosus (CLE) [138].

Similar to BDCA2, the ILT7 (also known as LILRA4) protein, which is exclusively expressed by pDCs, can also form a complex with FcRγ, and thereby leads to the inhibition of pDC functions. The cross-linking of ILT7 inhibited the production of IFNα in both CpG- and Flu-activated pDCs. The rapid phosphorylation of Src family kinases and Syk indicates that ILT7 cross-linking leads to the activation of ITAM mediated inhibitory signals in pDCs [105]. The bone marrow stromal cell antigen 2 (BST2), which is expressed only in low amounts on the surface of human pDCs, was identified as the biological ligand for ILT7. Following incubation with recombinant BST2, human pDCs were impaired in their ability to express type I IFNs upon challenge with CpG-A or Flu [139]. Interestingly, the Vpu HIV-1 protein hijacks this interaction by acting as an antagonist of BST2 and inhibiting TLR7-mediated type I IFN production by pDC. Vpu downregulates BST2 to enable virion assembly, while relocates remaining BST2 molecules to the surface of infected cells to maintain sufficient inhibitory signals for pDCs [140]. The ILT7-mediated negative feedback on type I IFN production is also hijacked by human cancer cells. Many human cancer cell lines constitutively express ILT7 ligands, thus inhibit TLR9-triggered IFNα production by human peripheral blood pDCs [141]. Interestingly, a study demonstrated that in vitro culturing of human peripheral blood mononuclear cells (PBMCs) leads to a spontaneous loss of ILT7 on the surface of pDCs. Consequently, the blocking of BST2 by mABs had no effect on the IFNα production of TLR7/9-triggered pDCs. Therefore, the authors propose that BST2-mediated ILT7 as a homeostatic regulator limits the activity of immature pDCs and does not serve as a negative feedback mechanism to restrict the functions of mature pDCs [142].

Another modulator of pDC functions is the transmembrane sialic acid binding immunoglobulin type lectins H (Siglec-H) receptor, which was identified as a specific marker of mouse pDCs. First it was demonstrated that cross-linking of Siglec-H with specific antibodies reduced TLR9-mediated type I IFN production of pDCs through the DAP12 adaptor protein [106]. In mice infected with murine cytomegalovirus (MCMV), Siglec-H deficiency induced elevated serum IFNα levels compared with wild type mice, whereas viral clearance was not affected [143]. Further, it was revealed that Siglec-H protects mice from developing MCMV virus-triggered lupus-like syndrome by preventing the induction of type I IFN signature [144].

According to a recent study, the Siglec-1 positive pDCs might represent the human counterpart of the Siglec-H positive pDCs in mice [107]. In humans, Siglec-1 is expressed in a subset of blood pDCs, which display a semi-mature phenotype, express lower levels of BDCA2 and interleukin (IL)-3 receptor α, and do not respond to TLR7/9 engagement. In vitro, its expression is inducible in Siglec-1 negative pDCs upon exposure of whole blood to Flu. Interestingly, the proportion of Siglec-1 expressing pDCs is higher in SLE patient and correlates with disease severity compared to healthy individuals; nevertheless, their functional role in SLE needs further investigations [107].

Another FcRγ-coupled regulator is the leukocyte mono-immunoglobulin-like receptor 8 (LMIR8), which is selectively expressed by mouse pDCs residing in the BM, spleen, or lymph nodes. It was found that LMIR8 cross-linking with mABs attenuates the CpG-A-mediated production of IFNα in BM-derived pDCs through the ITAM-containing adaptor protein FcRγ [108]. An additional pDC inhibitory receptor is NKp44, the expression of which is inducible on the surface of human tonsil and blood-derived pDCs upon culture with IL-3. It was reported that cross-linking of NKp44 inhibits IFNα secretion in CpG-activated pDCs through association with the ITAM-containing adaptor molecule DAP12 [109]. In contrast to NKp44, the expression of leukocyte-associated Ig-like receptor-1 (LAIR-1) is high on resting human pDCs, whereas it is downregulated in the presence of IL-3. The cross-linking of the inhibitory ITIM-containing LAIR-1 receptor impairs IFNα production by pDCs in response to TLR stimulation, and thus acts synergistically with Nkp44 to inhibit IFNα release [110]. Human pDCs also express the inhibitory C-type lectin receptor DC immunoreceptor (DCIR), which contains one ITIM motif. The cross-linking of DCIR inhibits TLR9-induced IFNα production, while it promotes antigen uptake and efficient antigen presentation by pDCs [112]. In mice, the ITIM-bearing paired immunoglobulin-like receptor B (PIR-B) is an inhibitory MHC-I receptor, which suppresses CpG-A triggered type I IFN production in mouse pDCs. In particular, PIR-B recruits the SH-2 domain-containing phosphatase 1 (SHP-1), which then leads to the dephosphorylation of STAT1/STAT2, and thereby prevents the autocrine type I IFN-mediated positive feedback loop [114].

In contrast to the aforementioned inhibitory receptors the following ones do not contain ITAM or associate with ITAM-containing adaptors, and the specific mechanism by which they regulate type I IFN responses of pDCs remained largely unknown. Protein tyrosine phosphatase receptor type S (PTPRS) is an evolutionarily conserved pDC specific inhibitory molecule, which is expressed by both murine and human pDCs, whereas protein tyrosine phosphatase receptor type F (PTPRF) is detectable only in mice. PTPRS cross-linking decreased type I IFN production in primary human pDCs and GEN2.2 cells upon CpG-A stimulation. In mice, quiescent pDCs co-express PTPRS and PTPRF, the knockdown of which enhanced TLR9-induced pDC activation. These receptors are downregulated on activated pDCs that seems to be a requirement for efficient type I IFN production. In addition, PTPRS and PTPRF deficiency was associated with enhanced pDC activation, increased leukocyte infiltration, and the development of spontaneous colitis in mice suggesting the importance of these receptors in maintaining immune homeostasis [115]. In addition, the Epstein-Barr virus induced receptor 2 (EBI2) functions as a negative regulator of type I IFN responses in pDCs as well. In mice, EBI2 is a chemotactic G protein-coupled receptor, which drives the migration of B cells and DCs through interaction with its ligand, 7α, 25-dihydroxycholesterol. In addition, it was found that EBI2 inhibits type I IFN responses of pDC upon stimulation with CpG-A, polyU or lymphocytic choriomeningitis virus (LCMV) through mechanisms depending on the Gαi subunit of the G protein [116].

Interestingly, CD28, the well-known costimulatory receptor of T cells, is also highly expressed on murine pDCs and acts as a negative regulator of type I IFN production. However, CD28 is undetectable in human blood circulating pDCs, it is expressed only by lymph node-derived human pDCs. BM-derived pDCs from CD28-deficient mice produce significantly higher levels of type I IFN compared to pDCs from wild type mice upon stimulation with TLR9 ligand, CpG-A or TLR7 ligand, Loxoribin. During MCMV or LCMV infection, systemic type I IFN levels were higher in CD28 knockout mice than in their wild type counterparts suggesting that CD28 controls TLR7- and TLR9-triggered IFN responses. Moreover, in a mouse wound healing model it was demonstrated that CD28 restricts IFN signature during non-viral innate immune responses as well [117].

In the previous section, we have established that in the course of inflammation HMGB1 binds to RAGE on the surface of pDCs and supports their type I IFN response. Paradoxically, in the tumor microenvironment, HMGB1 directly secreted by tumor cells renders pDCs tolerogenic. HMGB1 produced by neoplastic keratinocytes decreased IFNα secretion by human cord blood-derived pDCs following TLR9 stimulation. Moreover, pDCs co-cultured with neoplastic keratinocytes promoted the differentiation of naïve CD4+ T cells into regulatory T cells that could be reverted by the addition of the HMGB1 specific antibody [145]. One possible explanation for the inhibitory effect of HMGB1 in the tumor milieu might be that tumor-infiltrating pDCs highly express the T cell immunoglobulin and mucin domain-containing protein 3 (TIM-3), which binds HMGB1 with an affinity similar to that of RAGE and inhibits nucleic acid trafficking into endosomes leading to impaired TLR-mediated responses of mouse pDCs [118]. Specifically, treatment of tumor-associated mouse pDCs with mABs to TIM-3 resulted in a higher expression of IFNβ in response to CpG-A stimulation. Following stimulation with poly(dA:dT) dsDNA and HMGB1, it was observed that DNA uptake was much lower in the endosomal vesicles of TIM-3 expressing BM-derived mouse DCs compared to those from TIM-3 deficient mice [118]. These findings suggest that contrary to RAGE, TIM-3 serves as a negative regulator of endosomal TLR-mediated immune responses of DCs in the tumor microenvironment in a HMGB1 dependent manner (Table 2).

### 4.3. Receptors with Distinct Regulatory Roles: Fc Receptors

As we have described in the previous chapter, a number of ligand-binding regulatory receptors use the common γ subunit of FcRs as an interacting partner to manipulate the outcome of pDC responses. In addition, pDCs also express functional Fc receptors, which upon binding to their actual ligands, namely immune complexes or immunoglobulins, can send out activating or inhibitory signals, respectively [146].

PDCs have a predominant role in the pathogenesis of SLE, where immune complexes formed by self-nucleic acid and autoantibodies trigger pDC activation. It was recognized in the early 2000s that apoptotic cells in combination with serum IgG obtained from SLE patients can induce IFNα secretion by pDCs in PBMCs [147]. In a subsequent study, the authors also revealed that FcγRIIα (CD32), which is expressed on the surface of pDCs, is responsible for the increased IFNα secretion triggered by the combination of apoptotic cells and autoantibodies [148]. Later, it was also demonstrated that both necrotic and apoptotic cells release materials, namely RNA and DNA, which, in the presence of autoantibodies, induce the production of IFNα by human pDCs [149]. Thereafter, it was described that mouse pDCs lacking the common γ-chain of FcγR do not produce IFNα in response to DNA containing immune complexes. Further, it was found that FcγR-mediated internalization of DNA-autoantibody complexes is necessary to induce the non-canonical LC3-associated phagocytosis (LAP) pathway, which is required for TLR9 trafficking to phagosomes and initiation of IRF7-dependent IFNα secretion in mouse pDCs [119].

Similar to IgG, IgE in complex with self-DNA enhances type I IFN responses of pDCs as well. Following ligation, FcεRI, which is the major receptor mediating allergic inflammatory signals in mast cells, promotes the delivery of DNA to TLR9 and in this manner works in synergy with the FcγRIIα to activate pDCs. Intriguingly, IgE is much stronger in its ability to enhance the phagocytic potential of pDCs, probably due to its strong interaction with the high affinity FcεRI. By facilitating type I IFN secretion, self-reactive IgE exacerbates self-destructive autoimmune reactions, thus serum concentrations of DNA-IgE complexes correlate with disease severity in SLE [120].

In contrast, when not in complex with DNA, IgE suppresses pDCs in their ability to secrete IFNα in response to TLR9 triggering. A study showed that IgE cross-linking on pDCs with mABs reduces TLR9 expression and CpG-A-mediated secretion of IFNα by human pDCs [121]. Additionally, the authors later demonstrated that upon stimulation with anti-IgE antibody, pDCs secrete large amounts of tumor necrosis factor α (TNFα), which acts in an autocrine manner to reduce TLR9 on pDCs, and thus abrogates their type I IFN secretion [122]. Interestingly, a study showed that in allergic asthma patients, elevated serum IgE concentrations and increased surface FcεRI expression on pDCs correlate with their impaired antiviral response, which might contribute to disease exacerbation upon viral respiratory infections [150]. Nevertheless, it was recognized that omalizumab, the IgE neutralizing antibody used for the treatment of allergic asthma, restores the virus-induced IFNα responses of pDCs in allergic patients upon rhinovirus infection [151] implying further that free IgE has a negative impact on the type I IFN production of pDCs (Table 2).

### 4.4. Interactions of PRRs

As the first line of defense against infectious agents, the mammalian immune system expresses a myriad of PRRs, which recognize different conserved molecular structures known as pathogen-associated molecular patterns (PAMPs) and damage-associated molecular patterns (DAMPs). These receptors might interact with each other in either an inhibitory or a synergistic manner that might result in the modulation of host innate immune responses [152].

PDCs selectively express the endosomal TLR7 and TLR9, which might interact with each other during their activation. Interestingly, the presence of the TLR7 ligand R848 significantly decreased TLR9-mediated IFNα secretion in purified human pDCs by two different mechanisms [123]. On the one hand, R848 prevented the TLR9-mediated upregulation of IRF7 expression, and on the other hand it initiated TLR7 upregulation and TLR9 downregulation in pDCs. Both TLR7 and TLR9 are strong inducers of IFNα, thus the negative regulatory effect of TLR7 on TLR9-mediated signaling might have evolved as a compensatory mechanism to prevent the overproduction of type I IFNs that might be detrimental to the host [123]. Almost at the same time, another research group demonstrated that various natural and synthetic TLR7 ligands, even applied at substimulatory concentrations, significantly inhibited the TLR9-induced IFNα secretion in both human and mouse pDCs [124]. On the contrary, structurally different TLR7 ligands added simultaneously have a synergistic effect on pDC activity. In the human pDC cell line CAL-1, the co-administration of the single-stranded 9.2s RNA and the adenine analog CL264 markedly increased the secretion of IFNβ and pro-inflammatory cytokines through upregulating the expression of numerous immune-related genes. In addition, 9.2s RNA increased the cell surface binding, and thus probably the uptake of CL264 that might contribute to the synergistic effect of distinct TLR7 agonists on pDCs as well [153].

Besides TLRs, pDCs also express cell surface C-type lectin receptors, which recognize specific carbohydrate residues on bacterial and fungal cell surfaces. Specifically, the C-type lectin mannose receptor (MR) was found to synergize with the TLR9 receptor on pDCs. It was demonstrated that CpG-A in combination with the Cryptococcus neoformans-derived mannoprotein induced significantly more IFNα than CpG-A alone in mouse BM-derived pDCs. Furthermore, MR was found to synergize with other TLRs in conventional DCs as well, thus the study suggests that TLR ligands might be used as adjuvants in mannoprotein-based vaccines [125].

In addition, our research group observed that under steady-state conditions human pDCs express only marginal levels of cytoplasmic RIG-I receptor, an RLR family member, the expression of which can be greatly upregulated by TLR7/9 stimulation [40]. Later, we also demonstrated that melanoma differentiation-associated gene-5 (MDA5), another member of the RLR family, is also inducible upon the CpG-A treatment of human GEN2.2 pDC cells [126]. As we have described earlier in Chapter 2, RLR-mediated signaling contributes to the second wave of type I IFN production by pDCs, which shows the collaborative action of TLR and RLR families in the antiviral immune responses provided by pDCs [40].

Our research group recently demonstrated that two members of the nucleotide-binding domain leucine-rich repeat containing receptor (NLR) family, namely NLRX1 and NLRC5 inhibit the RIG-I and MDA5-mediated type I IFN responses of human pDCs. Silencing of NLRX1 and NLRC5 in GEN2.2 cells significantly increased the production of IFNα and IFNβ upon stimulation with synthetic RLR ligands or following infection with VSV. Thus, our findings suggest that these negative regulatory NLRs control antiviral signaling of pDCs to avoid aberrant IFN production and prevent the host from unwanted inflammation [126].

Lastly, it was demonstrated that stimulation of the DNA-sensing cyclic GMP-AMP synthase (cGAS)-stimulator of IFN gene (STING) pathway also interferes with the TLR9-mediated IFN production of human pDCs as well [127]. Both cGAS and STING are constitutively expressed by primary human pDCs and compared to TLR9 use a different cellular signaling machinery to induce type I IFN secretion. Interestingly, TLR9 and cGAS–STING signaling do not act synergistically, since pre-stimulation of the cGAS-STING axis markedly inhibited the type I IFN production of pDCs in response to CpG-A or HSV-1. The authors further revealed that cGAS–STING stimulation upregulated the expression of suppressor of cytokine signaling (SOCS) 1 and SOCS3, which might contribute to the downregulation of TLR9 signaling in pDCs [127] (Table 2).

The low frequency of primary pDCs in human blood sets a limit to the number of experiments, thus several receptors such as other cytosolic sensors wait for functional characterization, which could extend our knowledge regarding the receptor interactions regulating pDC type I IFN responses.

### 4.5. Adhesion Receptors

It has been long observed that pDCs form clusters upon in vitro culturing as well as in vivo in the T cell rich area of lymphoid tissues that seems to be critical for the production of optimal amounts of type I IFNs. At higher cell density, pDCs form clusters and spontaneously produce low amounts of type I IFNs, which then acts in a feedforward mechanism to amplify TLR-triggered type I IFN secretion [154].

Later, it was revealed that cell adhesion is required for TLR7 trafficking, since the lack of lymphocyte functional antigen 1 (LFA-1, also termed CD11a/CD18 or αLβ2) integrin decreased the ability of mouse pDCs to produce IFNα in response to TLR7 activation. Furthermore, it was found that LFA-1 activation induces microtubule polymerization that leads to TLR7 trafficking from endosomes to lysosomes, which enables the interaction with downstream signaling components such as TNF receptor associated factor 3 (TRAF) 3, IKKα, and mTOR required for IFNα/β induction. Moreover, the results suggest that cell adhesion-dependent type I IFN secretion boosts paracrine IFNα induction in pDC clusters [128].

Besides the above described homotypic cell adhesion, pDCs also interact with other cell types such as NK cells through LFA-1. NK cells promote the IFNα secretion of human pDCs stimulated with RNA-containing immune complexes through LFA-1 mediated cell–cell contact and macrophage inflammatory protein-1β (MIP-1β) production [155].

Furthermore, recent studies suggest that pDCs establish contact with infected cells through LFA1-dependent adhesion, which leads to the robust production of type I IFNs. Under in vivo conditions, the loss of the LFA-1 encoding gene impaired the type I IFN production of mouse pDCs upon CpG-A challenge or MCMV infection. Further, it was demonstrated that pDCs interact with infected cells through LFA-1 in the marginal zone of the spleen during MCMV infection that facilitates their type I IFN production [156]. These findings were further supported by another study showing that human pDCs interact with infected cells through the so-called interferogenic synapse, which enables the transfer of viral RNA to pDCs resulting in the local secretion of type I IFNs. In particular, initial encounter with virus infected cells activates TLR7 signaling in pDCs, which promotes structural reorganization including polarized endocytosis to facilitate the transfer of PAMPs to pDCs and sustained cell-cell contact through LFA-1, which further amplifies TLR7 mediated local secretion of type I IFN responses [157] (Table 2).

In summary, several cell surface and intracellular receptors control the type I IFN secretion of pDCs by sending out positive or negative feedback signals to fine-tune the amplitude of type I IFN response by pDCs. In the course of infection or inflammation a multitude of stimuli can trigger cells due to the distinct molecular structure of pathogens and release of multiple inflammatory factors that leads to the simultaneous activation of different receptors, and thus to the formation of a complex regulatory system.

## 5. Regulation of Type I IFN Production by Extracellular Soluble Factors

### 5.1. Tumor-Derived Factors

Tumors have the ability to modulate their microenvironment by secreting soluble factors, which can impair the capacity of tumor-infiltrating pDCs to secrete type I IFNs and eventually endows them with immunosuppressive properties [158].

The non-canonical wingless-related integration site 5a (Wnt5a) is a homolog of Wingless proteins in Drosophila species, which is secreted by cancer cells such as melanoma cells. Recombinant human Wnt significantly inhibited CpG-B triggered IFNα production by human pDCs, probably via inhibiting the cytoskeletal rearrangement required for cell activation [159]. In mouse splenic pDCs Wnt5a was found to upregulate the surface expression of indoleamine 2,3-dioxygenase-1 (IDO), which plays a significant role in DC tolerogenesis in melanoma [160].

The pleiotropic neuropeptide vasoactive intestinal peptide (VIP) is majorly released by neurons and immune cells [161]; however, a special type of neuroendocrine tumor is also able to secrete massive amounts of it [162]. A research group discovered that recombinant VIP decreases type I IFN production of primary human pDCs upon stimulation with CpG-A and CpG-B [163]. In a separate study, the authors further demonstrated that VIP effectively reduced TLR9-triggered IFNα secretion of pDCs from healthy individuals and from SLE patients as well [164].

Tumor cells can also secrete transforming growth factor β (TGFβ), which acting alone or synergistically with other cytokines also makes pDCs tolerogenic. The treatment of CAL-1 cells with tumor supernatant or combination of TGFβ and TNFα prevented TLR9-induced production of type I IFNs. On the one hand, TGFβ exposure sustains the expression of lysosome-associated membrane protein (LAMP5), which promotes TLR9 transport to late endosomes, and increases its degradation, thus limits type IFN secretion in pDCs. On the other hand, it is known that TNF promotes pDC maturation, and thus inhibits IFNα release [165]. Moreover, pDCs from breast tumors show a strongly upregulated expression of LAMP5 compared to pDCs isolated from the peripheral blood of the same patients [166]. A separate study also described that human tumor-associated pDCs purified from breast cancer samples secrete less IFNα compared to tonsil-derived pDCs from healthy controls in response to Flu and CpG-A [167]. Furthermore, tumor-derived TGFβ acts in synergy with TNFα to inhibit the ability of pDCs to produce IFNα via blocking IRF7 expression and nuclear translocation [168]. Similarly, pDCs purified from tumor samples of ovarian cancer patients also secrete lower amounts of IFNα compared to blood pDCs upon Flu and CpG-A stimulation due to the presence of TGFβ and TNFα [169]. TGFβ also suppresses CpG-induced IFNα production by purified tumor-associated pDCs in mouse models of lung cancer and melanoma [170]. Furthermore, pDCs from oral squamous cell carcinoma patients express lower levels of TLR9 and secrete less IFNα upon CpG-B stimulation. The oral cancer cell supernatant was observed to reduce TLR9 mRNA expression and inhibit TLR9-mediated IFNα production in human peripheral blood pDCs through a TGFβ and IL-10 dependent mechanism [171]. Another study showed that recombinant IL-10 can boost the suppressive effect exerted by the supernatants of different head and neck squamous cell carcinoma (HNSCC) cell lines on the IFNα secretion ability of pDCs [172]. Nevertheless, a recent paper reported that TNF rather than IL-10 is the major inhibitor of IFNα production by pDCs in HPV negative HNSCC, since neutralization of TNF in HNSCC supernatants almost completely restored the ability of pDCs to produce IFNα in response to CpG-A [173].

Moreover, TGFβ is also able to synergize with tumor-derived prostaglandin E 2 (PGE2) to abrogate IFNα production by purified human pDCs in response to different TLR7/9 stimuli [174]. TGFβ suppresses TLR-driven IFNα secretion, probably via inducing smad, the main signal transducers of TGFβ receptor, since blocking of smad abolished the inhibitory effect of TGFβ. In addition, PGE2 seems to exert its effect via the G protein-coupled PGE2 receptors, which upon ligand binding activate adenylate cyclase, and thereby increase the levels of cyclic adenosine monophosphate (cAMP), which is known to interfere with TLR-mediated signaling events [175]. Almost at the same time, another research group showed that prostaglandin analogs inhibited the IFNα secretion of TLR9-activated human pDCs with similar efficiency. Moreover, data suggest that PGE2 exerts its effect at the transcriptional level, since PGE2 significantly suppresses IRF7 mRNA expression in TLR9-stimulated PDCs [164].

Many tumor cells are also able to secret HMGB1, which, as an important component of the tumor milieu, plays a central role in cancer and metastasis development [176]. In the previous chapter, we mentioned that tumor-infiltrating pDCs start to express TIM-3, which upon interaction with HMBG1 interferes with the TLR-mediated immune responses of pDCs, and thus negatively influences their type I IFN secretion [118].

Another binding partner for TIM-3 is the conserved s-type lectin galectin-9 (Gal-9), which is a pleiotropic immune modulator. Besides TIM-3, Gal-9 has numerous binding partners and can be secreted by cancer cells via various mechanisms [177]. It has been recently recognized that Gal-9 inhibits the production of IFNα by mouse and human pDCs stimulated with various TLR7/9 ligands. Interestingly, it was found that the inhibitory effect of Gal-9 on the type I IFN secretion of pDCs is independent of TIM-3, and instead it is mediated through interaction with CD44 that leads to the disruption of the p70S6K/mTOR signaling pathway. Further, it was observed that Gal-9 administration suppressed the development of different autoimmune conditions in mice. In particular, it reduced inflammatory processes such as lymphocyte migration and alleviated disease symptoms such as splenomegaly in lupus prone mice [178] (Table 3).

### 5.2. Biogenic Amines and Steroid Hormones

Besides fulfilling their essential functions in the signaling events of the central and peripheral nervous system, biogenic amine neurotransmitters are also involved in the regulation of immune responses including the control of type I IFN production of pDCs [188,189].

Histamine, a major mediator of allergic diseases, was found to block IFNα release of human pDCs stimulated with CpG or Flu through the histamine receptor 2 (H2) [179]. Later, it was described that pDCs isolated from peripheral blood and from skin lesions of psoriasis patients express another histamine receptor subtype, termed H4, the expression of which can be further stimulated with CpG treatment [180]. Moreover, it was found that stimulation with H4 receptor agonists reduced the CpG-induced IFNα secretion similar to H2 receptor agonists. Interestingly, pDCs from psoriasis patients express higher H4 receptor levels, and thus show a stronger downregulation of IFNα secretion in response to H4 receptor agonists than pDCs from healthy individuals [180]. Another study reported that histamine can also efficiently block the IFNα secretion of HIV-1 activated purified human pDCs similar to other amines including dopamine and serotonin [181]. Further, the authors demonstrated that these amines exert their inhibitory effects on the antiviral capacity of pDCs through CXCR4 chemokine receptor engagement. In particular, natural amines do not act as ligands of CXCR4 but can interact with the receptor through their ammonium moiety and induce its internalization, which process seems to be essential for the inhibitory effects of amines [181].

Steroid hormones, due to their lipophilic nature, easily pass through plasma membranes, and by binding to soluble nuclear receptors are able to exert various effects on different immune cell types including pDCs [190]. A high number of findings indicate that estrogen plays an important role in the regulation of TLR-mediated responses of human and mouse pDCs as well. In mice, 17β-estradiol (E2) was described to significantly increase the IFNα production of CpG-B stimulated splenic pDCs [182]. In line with this, E2 treatment of postmenopausal women markedly elevated the TLR7/9-triggered IFNα production of primary pDCs. The authors further demonstrated that E2 directly targets pDCs, since deletion of estrogen receptor-α (ERα) abolishes the boosting effect of E2 treatment on the TLR induced IFNα production of mouse pDCs [183]. Furthermore, the impairment of estrogen receptor signaling significantly decreased the TLR7-induced IFNα expression in human pDCs generated from umbilical cord blood. [191]. Another study suggested that ERα signaling supports the IFNα secreting potential of TLR7-stimulated mouse pDCs by increasing the expression of IRF5, the level of which positively associates with the percentage of IFNα secreting pDCs. In addition, human pDCs overexpressing IRF5 exhibited increased IFNα secretion in response to TLR7 stimulation compared with control pDCs [184].

In contrast with estrogen, progesterone and its synthetic analogs have well documented immunosuppressive effects on innate immunity, and were observed to negatively regulate type I IFN secretion by human pDCs [192]. Under in vitro conditions, progesterone substantially inhibited the TLR9-mediated IFNα secretion of human and mouse pDCs. Moreover, the treatment of mice with depot medroxyprogesterone acetate (DMPA), an artificial progesterone significantly reduced the ability of isolated splenic pDCs to produce IFNα in response to CpG-A. Upon in vivo VSV infection, DMPA-treated mice had significantly lower serum IFNα levels compared with control mice. The authors further demonstrated that progesterone treatment inhibited the TLR9-induced nuclear accumulation of IRF7 in mouse BM-derived pDCs [185]. Another study showed that the synthetic progestin medroxyprogesterone acetate (MPA) is also able to impair the TLR7/9-stimulated IFNα production in human pDCs [186].

To date, only one study has investigated the effect of androgens on pDC function. The androgen hormone dihydrotestosterone (DHT) was demonstrated to decrease the TLR7-stimulated IFNα production of pDCs purified from the blood of healthy women. The authors further found that pDCs from male infants produced less IFNα in response to TLR7 stimulation compared to female infants that might be explained by the early postnatal androgen surge in male infants at 1–6 months of age [187]. In conclusion, while estrogen positively regulates the type I IFN release by pDCs, progesterone and testosterone affect it negatively.

The above data also indicate that gender differences greatly influence the individual immune responses to viral infections. Indeed, it is now widely accepted that gender-based immunological differences affect various aspects of immune responses including the susceptibility to infections, the prevalence of autoimmune diseases, and the efficacy of vaccination [193]. Moreover, pDCs are highly sensitive to their environment and the myriad of soluble factors released from different cell types can highly impact the intensity of their type I IFN responses (Table 3).

## 6. Targeting Type I IFNs in pDC-Related Pathological Conditions

### 6.1. Viral Infections

The rapid and robust type I IFN production of pDCs is essential to control the early phase of viral infections by directly inhibiting viral replication, preventing virus-triggered tissue damage, and activating a wide repertoire of cellular and humoral innate immune signaling elements. The important role of pDCs in overcoming viral infections has already been highlighted in different mouse models upon mouse hepatitis virus (MHV), HSV, dengue virus (DENV), chikungunya virus (CHIKV), LCMV and respiratory syncytial virus (RSV) infections (reviewed in [1]).

However, if the immune system is not able to effectively clear the virus, it leads to the development of chronic infections associated with persistent inflammation and immune activation, in which the adverse effects of type IFNs are manifested. Furthermore, chronic viral exposure negatively affects pDC functions. For instance, HIV positive patients with high viral load are characterized by functionally exhausted pDCs with decreased type I IFN secretion and increased rate of apoptosis, which leads to alterations in pDC population dynamics [194]. Nevertheless, type I IFNs released by pDCs play a versatile role in the course of HIV-1 infection. At the early phase of infection, the secreted large amounts of type I IFNs have beneficial effects, whereas during the late phase, chronic activation of pDCs results in type I IFN-driven pathologies such as T cell exhaustion, apoptosis of uninfected CD4+ T cells through an increased expression of programmed cell death 1 (PD-1) receptor as well as generation of regulatory T cells via upregulating tolerogenic mediators such as IL-10 or IDO [195,196,197,198]. Based on these data, the clinical usage of type I IFN therapy in HIV-1 infection is quite controversial, since it seems to be beneficial in the acute phase, whereas it is not advised in the chronic phase of infection [199,200]. In spite of this, it is important to note that recombinant human IFNα2b is indicated for the treatment of various human chronic viral infections associated with malignant conditions such as acquired immune deficiency syndrome (AIDS)-related Kaposi’s sarcoma, human papilloma virus (HPV)-caused condyloma acuminata, and chronic hepatitis C (HCV) or hepatitis B (HBV) infections [201,202,203,204].

The novel coronavirus pandemic caused by the severe acute respiratory syndrome coronavirus 2 (SARS-CoV-2) strain, further emphasizes the importance of the timing of type I IFN therapy in the treatment of viral infections. So far, a rapidly growing number of publications have focused on the association between type I IFNs and coronavirus disease 2019 (COVID-19) [205,206,207,208,209]. The progression of COVID-19 can be divided into 3 stages [210]. The first phase of the disease is asymptomatic or characterized by mild flu-like symptoms, which is followed by the second pulmonary phase designated by viral pneumonitis. In some individuals, the infection culminates further in the last and mostly lethal hyperinflammation phase, which results in shock or acute respiratory distress syndrome (ARDS) due to the dysregulated antiviral and pro-inflammatory response, known as a “cytokine storm” [210]. It is worth noting that SARS-CoV-2 uses distinct strategies to bypass the early type I IFN response of the host, and due to the delayed antiviral response the viral load gradually increases that eventually leads to the induction of cytokine storm and recruitment of inflammatory immune cells to the lung [205]. Thus, in COVID-19 patients, decreased production of antiviral type I IFNs and large amounts of pro-inflammatory mediators, primarily TNFα, ILs (e.g., IL-1β, IL-2, IL-6, IL-7, IL-10, IL -12, IL-18, IL-33) and chemokines can be observed [211]. It was also found that factors influencing type I IFN production, such as sex, age, genetic defects in IFN signaling, or autoantibodies against type I IFNs, predispose some to more severe disease outcomes highlighting the protective role of these antiviral cytokines in the early phase of infection [68,208,212]. Although pDCs do not express the angiotensin converting enzyme 2 (ACE-2) entry receptor of the virus and are resistant to active infection, it seems that they can be fully activated when challenged with SARS-CoV-2 strains. However, the data so far implicate that pDCs are not the culprit behind the fatal cytokine storm developing in some patient with severe COVID-19 [213].

Thus, the above data arise the question, whether type I IFN administration might have therapeutic benefits in the treatment of COVID-19 patients. Previous experiments with SARS-CoV or Middle East respiratory syndrome coronavirus (MERS-CoV) showed that IFN therapy is only effective when it is used as prophylaxis or in the initial phase of the infection, whereas at later stages, type I IFNs can be ineffective or even detrimental to the host [205,207]. Intranasally administered type I IFNs can be used as a prophylaxis to prevent SARS-CoV-2-triggered symptoms and exogenous type I IFNs might also help to clear the infection after SARS-CoV-2 reached the lung. Nevertheless, in order to avoid the exaggeration of inflammation type I IFN administration is not recommended in the third stage of the disease [206,207,211,214]. Currently, a number of possible immunotherapeutic approaches including the targeting of type I IFN responses are under evaluation for the treatment of COVID-19, which are extensively reviewed in reference [215].

**Figure 2:**
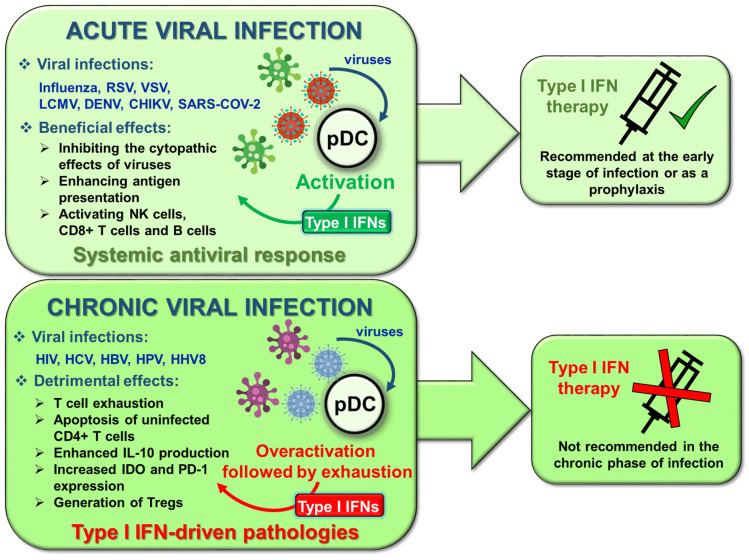
**The Janus-faced role of pDCs in viral infections.** Via producing large amounts of type I IFNs, pDCs are essential to initiate an effective antiviral response. Recombinant type I IFN therapy is suggested for the treatment of acute viral respiratory tract infections or as a prophylaxis (**upper panel**). On the contrary, persistent and uncontrolled activation of pDCs leads to type I IFN-driven pathologies upon chronic viral infections. Therefore, the use of type I IFNs is not recommended for the treatment of chronic viral infections as it may exacerbate pre-existing inflammation (**lower panel**). *CHIKV: chikungunya virus; DENV: dengue virus; HBV: hepatitis B virus; HCV: hepatitis C virus; HHV8: human herpesvirus 8; HIV: human immunodeficiency virus; HPV: human papillomavirus; IDO: indoleamine-2,3-dioxygenase; IFN: interferon; IL: interleukin; LCMV: lymphocytic choriomeningitis virus; NK: natural killer; PD-1: programmed cell death protein 1; pDC: plasmacytoid dendritic cell; RSV: respiratory syncytial virus; SARS-COV-2: severe acute respiratory syndrome coronavirus 2; VSV: vesicular stomatitis virus.*

Overall, in viral infections, type I IFN therapies might be indicated at the early stage of infection to avoid uncontrolled viral replication and the cytopathic effects of the viruses; however, due to their strong ability to activate immune cells, exogenous type I IFNs are no longer recommended for the treatment of infections associated with extensive inflammation (Figure 2).

### 6.2. Cancer

Besides viral infections, type I IFNs might be promising therapeutic targets in cancer, since type I IFNs possess direct as well as indirect antitumor effects owing to their ability to control the activation of innate and adaptive immune cells, protein synthesis, autophagy, apoptosis, and angiogenesis. [216]. It is noteworthy that the first cancer immunotherapy approved by the Food and Drug Administration (FDA) was recombinant IFNα therapy; however, the development of severe systemic autoimmune reactions was observed in certain individuals as a side effect [217]. Although, nowadays many other drugs with less severe side effects are available on the market, type I IFNs might be back in the game, since many on-going clinical trials combine type I IFN-based strategies with other treatment protocols to develop more efficient therapies for cancer treatment [218].

The infiltration of pDCs has been reported in the microenvironment of many different types of tumors such as in the stroma of melanoma, ovarian carcinoma, breast cancer, glioma, head and neck tumors, colorectal carcinoma, lung cancer, and hepatocellular carcinoma [219,220,221], where they usually display a tolerogenic phenotype and support tumor growth. Thereby their accumulation is associated with poor prognosis in breast cancer, ovarian cancer, oral squamous cell carcinoma and melanoma [169,222,223,224,225,226,227]. In the tumor niche, a large number of tumor-derived factors contribute to the reprogramming of infiltrating pDCs, which develop an immature, tolerogenic phenotype with impaired IFNα production, and are involved in the maintenance of an immunosuppressive tumor microenvironment. Therefore, the re-activation of tumor-associated pDCs would be essential to the development of an effective anti-tumor response, which makes pDCs promising targets in anti-tumor therapies. Activated pDCs have direct cytotoxic effects by inducing granzyme- and TRAIL-dependent apoptosis of tumor cells [228,229,230]. In addition, they can also exert indirect anti-tumor effects by producing IFNα, which effectively activates the anti-tumor response of NK cells and CD8+ T cells [231]. Thus, in cancer treatment one of the major therapeutic goal is the re-activation of tumor-associated pDCs that could help to revive their beneficial direct and indirect anti-tumor activities.

Among the different types of cancer therapies targeting the pDC-type I IFN axis has potential benefits in the treatment of metastatic melanomas. To boost pDC-mediated anti-tumor responses, specific TLR7 and TLR9 agonists can be applied as a monotherapy or in combination with other anti-tumor agents. For example, topically applied imiquimod in combination with monobenzone resulted in the local regression of cutaneous metastases in half of the melanoma patients [232]. Imiquimod has also been used as a vaccine adjuvant for the immunization of malignant melanoma patients [233]. In addition, TLR9 agonists as monotherapy or in combination with checkpoint inhibitors also represent possible therapeutic options [234,235,236]. Furthermore, pDC-based vaccines have also been tested to utilize the cross-presentation capacity of activated pDCs. In a study, autologous pDCs isolated from the peripheral blood of melanoma patients were activated and loaded with tumor-associated peptides, then injected into the lymph node of the patients [237]. The vaccine was able to elicit the systemic secretion of type I IFNs and induction of tumor antigen-specific CD8+ T cells [237]. Importantly, HLA matched allogeneic pDCs loaded with melanoma-derived antigens are also able to effectively activate tumor-specific T cells that opens up new avenues for their usage in adoptive cellular immunotherapy [238,239]. The main approaches targeting pDCs in melanoma including clinical trials are summarized in detail in a recent review [220].

The potential of targeting the pDC-type I IFN axis was also explored in other malignancies. The efficiency of IFNα with combined checkpoint inhibition was tested in metastatic melanoma and renal cell carcinoma as well; however, the combination therapy was discontinued due to the poor tolerability and low antitumor activity [240]. In a phase 1/2 study, intratumoral CpG injection in combination with radiotherapy elicited a clinically meaningful response in patients with low-grade B cell lymphoma [241] and mycosis fungoides [242]. Furthermore, a vaccination of pDCs and/or myeloid DCs loaded with peptide antigens evoked functional antigen-specific T cell responses in patients with chemo-naive castration-resistant prostate cancer, thus it might be a promising immunotherapy approach to treat prostate cancer [243]. Currently, there are ongoing clinical trials using pDC-based vaccines for the treatment of metastatic endometrial cancer and non-small-cell lung cancer as well [244].

**Figure 3:**
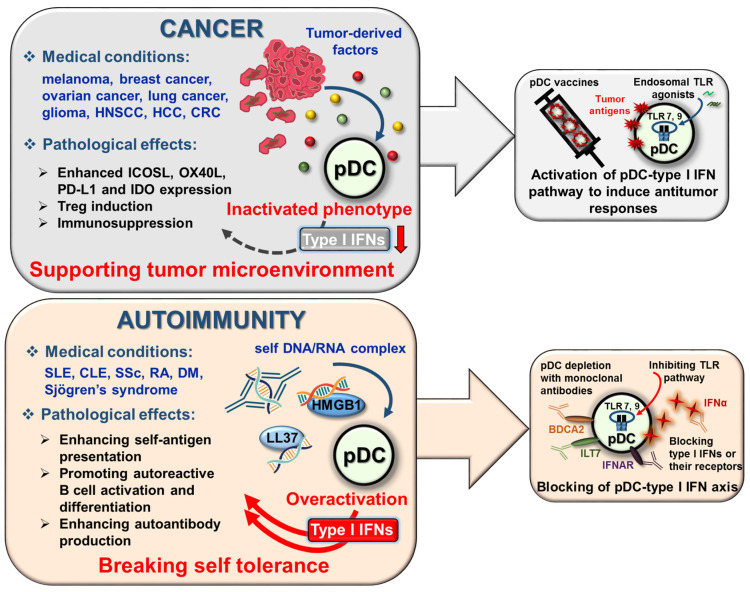
**pDC-type I IFN axis is implicated in the pathogenesis of cancer and autoimmunity.** Due to the suppressive tumor-derived factors, pDCs are the “sleeping beauties” of tumor microenvironments, and thus are unable to use their valuable anti-tumor activity, which further supports tumor growth. Therefore, reawakening of pDCs with different endosomal TLR ligands might elicit their direct and indirect type I IFN-dependent antitumor responses. Furthermore, tumor antigen-loaded pDCs represent promising vaccine candidates as well (**upper panel**). On the contrary, overactivation of pDCs maintains a prolonged interferon gene signature (IGS) and fuel autoimmunity. Thus, in the therapy of pDC-associated autoimmune diseases the main goal is to reduce the activity of the pDC-type I IFN axis using monoclonal antibodies, which deplete or inhibit pDCs, neutralize circulating IFNα or block IFNAR receptors. TLR7/9 antagonists are also undergoing trials in the treatment of these disorders (**lower panel**). *BDCA2: blood dendritic cell antigen 2; CLE: cutaneous lupus erythematosus; CRC: colorectal cancer; DM: diabetes mellitus; HCC: hepatocellular carcinoma; HMGB1: high mobility group box protein 1; HNSCC: head and neck squamous cell carcinoma; ICOSL: inducible T cell costimulator ligand; IDO: indoleamine-2,3- dioxygenase; IFN: interferon; IFNAR: interferon-alpha/beta receptor; ILT7: immunoglobulin-like transcript 7; OX40L: OX40 ligand; pDC: plasmacytoid dendritic cell; PD-L1: programmed cell death protein 1; RA: rheumatoid arthritis; SLE: systemic lupus erythematosus; SSc: systemic sclerosis; TLR: Toll-like receptor; Treg: regulatory T cell*.

Based on the anti-tumor properties of pDCs, which are partially provided by their type I IFN production, re-activation of pDCs and restoring their type I IFN producing capacity might offer a promising adjuvant therapy to enhance the efficacy of conventional cancer treatments (Figure 3).

### 6.3. Autoimmunity

In the last decade, type I IFN pathway emerged as an important therapeutic target for the treatment of type I IFN-driven autoimmune diseases; however, the success rates of clinical trials are varying (10.1136/lupus-2019-000336). Autoimmune conditions are commonly accompanied by IGS, which refers to the increased expression of genes regulated by type I IFNs. IGS can be observed in the blood and/or in the affected inflamed tissues of patients with CLE, SLE, dermatomyositis, RA, systemic sclerosis (SSc) and Sjögren’s syndrome [10,245,246,247]. Autoimmune reactions can be associated with over-activated pDCs, which are rapidly recruited to inflamed tissues, where they actively produce type I IFNs and interact with other immune cells to exacerbate inflammation. For instance, it was described that pDCs promote plasmablast differentiation and autoantibody production in SLE through the release of IFNα and CD40 engagement [248]. Since a clear association exists between infiltrated pDC numbers and type I IFN overproduction, it seems feasible that the selective targeting of pDCs or their signaling molecules would be a more potent approach to control excessive type I IFN production in these disorders [249].

Autoimmune diseases are currently incurable conditions, and most therapies are based on non-specific immunosuppression using glucocorticoids or cytostatic agents to reduce the severity of symptoms [249]. These therapeutic agents also act on pDC functions, for example, steroid administration decreases pDC numbers and their ability to produce type I IFNs in SLE patients. However, after discontinuing glucocorticoids, both the number of pDCs and the level of IFNα recovered rapidly [250,251]. Hydroxychloroquine (HCQ) can also diminish type I IFN production by TLR7 or TLR9 activated pDCs from SLE patients [252]. Another study found that clinically relevant high serum HCQ levels reduced TLR9 but not TLR7/8 induced type I IFN production in pDCs from CLE patients [136]. Furthermore, mycophenolic acid, the active form of mycophenolate mofetil used to treat lupus nephritis is also able to dose-dependently suppress CpG-induced type I IFN secretion from pDCs of SLE patients via inhibiting nuclear translocation of IRF7 [253]. Similarly, clinically relevant doses of arsenic trioxide, which is used to treat SSc, could also diminish type I IFN release by CpG-A activated pDCs through downregulating the expression as well as the phosphorylation of IRF7 [254].

A more specific approach to treat autoimmune diseases is using monoclonal antibodies to block the actions of over-activated cells or to reduce the level of factors associated with disease exacerbation [249]. With regard to pDCs, clinical trials mainly target IFN signaling downstream of pDCs by using monoclonal antibodies, which neutralize IFNα or block type I IFN receptors [255,256]. In addition, novel approaches are focusing on the depletion or functional inhibitions of pDCs. For example, the ILT7 targeting antibody MEDI7734 (alternative name: VIB7734) is in a phase I clinical trial for the treatment of various type I IFN-mediated autoimmune diseases, whereas the BDCA2 targeting BIIB059 is in phase II clinical trial for the treatment of SLE and CLE [136,137,257]. Moreover, inhibitory molecules blocking the endosomal TLR signaling pathway such as the IRAK-4 inhibitor PF-06650833 are also in clinical trials for the treatment of rheumatic and autoimmune diseases [258]. A promising approach for the treatment of IGS-associated diseases is active immunization with IFNα-kinoid, a therapeutic vaccine consisting of inactivated recombinant human IFNα2b and a carrier protein, the keyhole limpet haemocyanin, which can induce a strong polyclonal antibody response against IFNα and thus reduce IGS in patients with SLE [259,260]. Clinical trials focusing on the pDC-type I IFN axis are reviewed in more details in references [196,249].

Overall, as opposed to anti-tumor therapies, the goal of non-specific and antigen-specific immunotherapies in the treatment of IGS-associated autoimmune conditions is to reduce excessive type I IFN production, which can improve the life quality of patients (Figure 3).

### 6.4. Allergy

Besides the aforementioned pathological conditions, pDCs and their type I IFN production have also been implicated in the pathogenesis of allergic diseases. Studies found that pDCs are present in the nasal mucosa of allergic patients [261,262], and after allergen inhalation their number increases in the airways [263,264]. However, in allergic skin lesions, the number of pDCs may vary depending on the type of hypersensitivity reaction and associated cytokine milieu, which can affect their viability in the lesional skin [265,266].

Among allergic diseases, the role of pDCs has been most extensively studied in asthma, a chronic inflammatory disease of the airways, and it seems that due to their tolerogenic functions, pDCs help to relieve clinical symptoms and have a protective effect on disease progression. In allergy, type I IFN production could account for the beneficial effects of pDCs, since type I IFNs have the ability to inhibit the development of Th2 cells, the functions of innate lymphoid cells 2 (ILC2), the secretion of Th2 cytokines, and the isotype switching to IgE. Besides, pDCs drive regulatory T cell expansion and thus contribute to the induction of tolerance in allergic diseases [267]. However, asthmatic patients are characterized by attenuated type I IFN production and uncontrolled Th2 response that makes them more susceptible to viral infections and infection-induced asthma exacerbations. The impaired type I IFN production might be the result of FcεRI cross-linking, which negatively effects the type I IFN pathway [268,269,270].

Multiple studies have shown the tolerogenic potential of pDCs in different mouse models of allergic airway inflammation [271,272,273,274]. In a recent study, it was shown that neonatal mice are more susceptible to severe allergic airway inflammation compared to adult mice that could be associated with the lower percentage of pDCs and thus reduced level of IFNα in neonates. Nevertheless, adoptive transfer of pDCs or addition of IFNα to neonatal mice inhibited the development of allergic inflammation. Depletion of pDCs in adult mice resulted in more severe allergic inflammation as well. The authors also reported that the protective effects of pDCs are mediated through their IFNα release, which inhibits the secretion of epithelial cell-derived chemotactic factors and thus the recruitment of Th2 promoting cDCs and ILC2 cells. In line with that, the percentage of pDCs and levels of IFNα were lower in the sputum of asthmatic children than in adult patients that might render children more susceptible to viral infection [275]. Indeed, reports suggest that lower frequencies of circulating pDCs correlate with the risk of lower respiratory tract infections in infants and young children up to the age of 5 years [276,277].

Despite their beneficial effects in preventing allergy development at early ages, studies with experimental animal models suggest that pDCs drive virus-induced asthma exacerbation when the disease is already established. After rhinovirus infection of mice with allergic airway disease pDCs were recruited to the lung then migrated to the draining lymph nodes to trigger Th2-mediated allergic responses, which could be abrogated by pDC depletion. Consistent with that, higher pDC levels were detected in the sputum of asthmatic patients during acute exacerbations, and their numbers correlated with the severity of inflammation and frequency of asthmatic attacks [278].

The above data indicate that due to their tolerogenic properties pDCs contribute to the maintenance of a healthy Th1/Th2 balance, thus adequate pDC numbers and functions have a protective effect on the development of allergic diseases. Nevertheless, when the allergic disease is already established, acute viral infections might trigger the inflammatory properties of pDCs, which negatively affects the allergic condition and causes the exacerbation of symptoms. However, it is worth to mention that while the protective role of the pDC-type I IFN axis is well established in infants and children with asthma, there is controversy regarding the role of pDCs in adult asthmatic patients. Therefore, further studies are needed to define the exact function of pDCs in the context of asthma and to resolve these contradictions [279].

**Figure 4:**
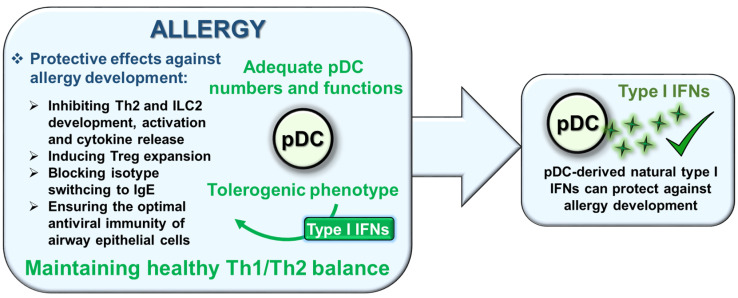
**Role of pDC-type I IFN axis in allergy.** PDCs have a protective role in the development of allergic diseases via secreting type I IFNs, which help to maintain a healthy Th1–Th2 balance and prevent the shift towards Th2 dominance in the airways. *IFN: interferon; IgE: immunoglobulin E; ILC2: type 2 innate lymphoid cells; pDC: plasmacytoid dendritic cell; Th: T helper; Treg: regulatory T cell.*

Inhaled corticosteroids are commonly used for the long-term treatment of asthmatic patients to resolve symptoms. However, it is important to note that these drugs can suppress type I IFN production and contribute to the onset of a Th2-dominant phenotype that makes patients more vulnerable to respiratory tract infections [270]. Therefore, type I IFN therapy could be utilized for the treatment of patients with allergic asthma and other chronic atopic diseases with Th2 dominance, in which type I IFNs could counterbalance Th2 cell-mediated inflammation. Preclinical and clinical trials using type I IFN-based therapy for the treatment of atopic diseases are summarized in references [268,270] (Figure 4).

## 7. Discussion

Type I IFNs are one of the most potent soluble mediators of viral infections, which serving as a universal alarm signal are able to alert each cell in the body to establish an antiviral state and fight against viruses [14]. Therefore, all of our nucleated cells ubiquitously express type I IFN receptors and are able to produce type I IFNs themselves that contribute to the formation of a complex cell-to-cell communication network, and thus to the generation of an efficient antiviral response [14]. PDCs are specialized in the production of type I IFNs, and since they are predominantly located in the lymph nodes in the steady-state [280], the cytokines released by them in response to viral infections can reach all cells through the lymphatic and blood circulation system thereby ensuring a systemic effect. Since pDCs are able to rapidly produce massive amounts of type I IFNs, a tight spatiotemporal regulation is needed to utilize their beneficial effects and avoid their damaging activities. Upon infections, multiple stimuli can simultaneously trigger pDCs resulting in the activation of parallel signaling pathways, which upon interaction orchestrate the type I IFN production of pDCs. In addition, a myriad of adaptor proteins and intracellular regulatory molecules are also involved in the control of type I IFN secretion by pDCs (Table 1). To support the rapid and extensive production of type I IFNs by pDCs, adaptor proteins positively regulate the transcription factors, which are essential to the induction of type I IFNs (Table 1). Nevertheless, in order to limit type I IFN-driven host tissue-damage a vast number of receptors initiate negative feedback mechanisms to fine-tune the length and amplitude of type I IFN responses (Table 2). Moreover, the changes of microenvironment and the cell- or tissue-derived factors can also significantly influence the magnitude of type I IFN responses by pDCs (Table 3). Besides microenvironmental factors, the autocrine type I IFN signaling loop expands further the list and enhance the complexity of the regulatory system [14].

It is also important to note that the human body prepares for viral attacks prior to viral exposure by providing tonic type I IFN signaling. Under physiological conditions, various cell types and tissues can continuously produce basal levels of type I IFNs that are crucial to an effective antiviral response. In addition, they contribute to the maintenance of immune homeostasis and the regulation of numerous physiological processes as well [25]. It is especially important to maintain low-level constitutive IFN signaling at mucosal surfaces, the major entry sites for viruses that is driven by commensal microbiota-derived tonic signals [281]. The importance of IFN-primed state is also supported by literature data showing that the improper use of antibiotics might predispose a patient to more severe flu symptoms due to antibiotic-induced damage of the microbiota, which ultimately leads to decreased antiviral activity of epithelial cells [282]. In addition to the fact that each individual has a unique microbiome, several other factors such as gender related differences in immune responses, genetic defects in type I IFN signaling, various pathological conditions, drugs, or biological therapies can also influence the individual’s baseline IFN signature and antiviral capacity. Thus, the severity of viral infections might vary greatly among individuals even though they are infected by the same virus [193,283].

For instance, females mount a stronger and more effective humoral and cellular immune response than males due to the positive stimulatory effect of the female sex hormone, estrogen, which also supports the type I IFN production of pDCs [183,190]. In addition to sex hormones, X chromosome dosage can also influence the IFN response of pDCs. In a humanized mouse model, where CD34+ human progenitor cells (HPC) were transplanted into female or male mice, pDCs developed from female HPCs produced higher amounts of type I IFNs in response to TLR7 stimulation as compared with pDCs from male donors regardless of the sex of the recipient mice. These data imply that the double X chromosome in females has an immunological advantage by contributing to an enhanced immune response against infections. [191]. A similar study also investigated the effect of X chromosome number and sex hormones on the TLR7-mediated IFNα production by primary human pDCs from healthy females, males, transgender volunteers on hormone therapy, and females with Turner syndrome. The authors found that the expression of the type I IFN-inducible antiviral membrane protein tetherin and the TLR7-induced IFNα production are higher in females that correlates with the number of X chromosomes but not with sex hormone serum levels [284]. It has been long proposed that several genes involved in TLR signaling might escape from X inactivation and account for the strong immune responses in females [285]. Indeed, single cell analyses of TLR7 allelic expression in pDCs, B cells and monocytes from females and Klinefelter (XXY) individuals revealed that TLR7 escapes from X chromosome silencing. A recent study further demonstrated that female pDCs with biallelic TLR7 expression have higher type I IFN production than monoallelic TLR7 expressing pDCs, presumably due to the IFNAR mediated type I IFN positive feedback loop [286]. This molecular mechanism might also provide a plausible explanation for the higher COVID-19 mortality rates among males compared to females [287,288]. The above data suggest that female pDCs with biallelic TLR7 expression respond more rapidly to SARS-CoV-2 infection by producing large amounts of type IFNs that might account for the better control of SARS-CoV-2 infection in women compared to men [286]. This idea is also supported by a recent report showing that loss-of-function mutation of X-chromosomal TLR7 is associated with impaired type I IFN responses of young male patients with severe COVID-19 that further emphasizes the importance of intact TLR7-mediated type I IFN responses in the pathogenesis of the disease [289]. Moreover, pDC-derived type I IFNs mediate B cell activation and differentiation into plasma cells, thus are essential to elicit an optimal antibody response to viral infection including SARS-CoV-2 infection as well [290].

All the above data highlight the critical importance of type I IFNs in mounting an effective antiviral response. Notably, immunosuppressive drugs such as corticosteroids, which are commonly used in the therapy of autoimmune and allergic diseases, inhibit type I IFN signaling, and thus enhance the susceptibility of patients to viral infections [251,291]. Furthermore, it should be noted that type I IFN therapies might have undesired outcomes when the timing and dosage is not adjusted properly. For instance, type I IFN therapy can be used as prophylaxis to reduce the severity of acute viral infections; however, its usage is not recommended during chronic viral infections, since it would exacerbate disease symptoms [292]. Furthermore, type I IFNs are used for the treatment of cancer due to their tumor suppressive activity; nevertheless, they might induce robust autoimmune adverse events [218].

Due to their high plasticity, pDCs are able to rapidly switch their phenotype and function in response to microenvironmental changes, and acquire either immunogenic or tolerogenic properties; thus, different disease-causing phenotypes might arise depending on the associated pathological condition [293]. PDCs are unique in many of their properties among immune cells and differ from their conventional counterparts in multiple aspects including their receptor pattern, phenotypic and metabolic profile as well as their effector functions [294]. Therefore, regulatory mechanisms, which have been widely explored in cDCs cannot be extended to pDCs. Without doubt, pDCs are professional in type I IFN production, and thus play a prominent role in antiviral responses; however, their inadequate or excessive type I IFN production might play a key role in the pathogenesis and pathomechanism of several human diseases. Although, currently, several ongoing clinical trials target the pDC-type I IFN axis for treating pDC-associated diseases, further investigations are needed to fully explore the mechanisms regulating the type I IFN responses by pDCs that might help to better understand the complex regulation of type I IFN production, and thus might improve the efficacy of these therapies.

## Figures and Tables

**Table 1:** Regulation of type I IFN production at the transcriptional and posttranscriptional level.

Transcription Factors				
**Regulating Factor**	**Type of Regulation**	**Mechanism of Regulation**	**Model**	**Ref.**
IRF5	positive	induces the expression of type I IFN genes	mouse	[46,47]
IRF5	positive	induces the expression of type I IFN genes	human	[48]
IRF8	negative	inhibits IRF5	human	[48]
IRF8	positive	-	mouse	[49]
RUNX2	positive	induces IRF7 expression	human	[50]
Spi-B	positive	transactivates the promoters of type I IFNs	mouse	[51]
NFATC3	positive	binds to type I IFN promoters in synergy with IRF7 (mechanism demonstrated on human pDCs)	mouse/ human	[52]
MYC	negative	represses IRF7 promoter activity	human	[53]
CXXC5	positive	maintains constitutive transcription of IRF7 (mechanism demonstrated on mouse pDCs)	mouse/ human	[55]
E2-2	positive	supports the expression of TLR7, TLR9, IRF7, IRF8 and Spi-B	mouse	[56]
E2-2	positive	downregulates the expression of TLR10 and Siglec-6	human	[57]
**Adaptor Proteins and Other Intracellular Regulators**				
**Regulating Factor**	**Type of Regulation**	**Mechanism of Regulation**	**Model**	**Ref.**
Opn-i	positive	supports the nuclear translocation of IRF7	mouse	[58]
PACSIN1	positive	-	mouse/ human	[59]
TRIM8	positive	prevents phosphorylated IRF7 from proteasomal degradation (demonstrated on HEK293T)	human	[60]
PLSCR1	positive	supports TLR9 trafficking to the early endosomes (mechanism demonstrated on human pDCs)	mouse/ human	[61].
SphK1	positive	regulates the nuclear transport of IRF7 and uptake of CpG (mechanism demonstrated on human pDCs)	mouse/ human	[62]
SCARB2	positive	mediates TLR9 trafficking and the nuclear translocation of IRF7	human	[63]
mTOR	positive	supports TLR-mediated IRF7 phosphorylation and nuclear translocation	mouse/ human	[64]
mTOR	positive	supports RLR-mediated TBK1 phosphorylation	human	[65]
mtROS	negative	suppresses TLR9-triggered type I IFN production through inhibiting IRF7 phosphorylation	human	[66]
mtROS	positive	supports RLR-triggered type I IFN production through IRF3 phosphorylation	human	[66]
ROS	negative	inhibits TLR7-mediated type I IFNs	human	[67]
**MicroRNAs**				
**Regulating Factor**	**Type of Regulation**	**Mechanism of Regulation**	**Model**	**Ref.**
miR-155	negative	represses TAB2	human	[68]
miR-155 *	positive	suppresses IRAK-M	human	[68]
miR-146a	negative	targets IRAK-1	human	[69]
miR-618	positive	-	human	[70]
miR-21	positive	suppresses PTEN	mouse	[71]
miR-126	positive	targets TSC1	mouse	[72]

*Abbreviations: CXXC5: CXXC-type zinc finger protein 5; IFN: interferon; IRAK: interleukin 1 receptor associated kinase 1; IRF: interferon regulatory factor; miR: microRNA; mtDNA: mitochondrial DNA; mTOR: mammalian target of rapamycin; mtROS: mitochondrial ROS;NFATC3: nuclear factor of activated T cells 3; Opn-i: intracellular osteopontin; Ox-mtDNA: oxidized mitochondrial DNA; PACSIN1: protein kinase C and casein kinase substrate in neurons 1; PLSCR1: phospholipid scramblase 1; PTEN: phosphatase and tensin homolog; RLR: RIG-I-like receptor; RUNX2: Runt-related transcription factor 2; SCARB2: scavenger receptor class B member 2; SphK1: sphingosine kinase 1; TAB2: TGFβ activated kinase 1 binding protein 2; TBK1: TANK-binding kinase 1; TLR: toll-like receptor; TRIM8: tripartite motif containing protein 8; TSC1: tuberous sclerosis complex 1.*

**Table 2:** Regulation of type I IFN production by receptor interactions.

Activating Receptors of Type I IFN Production				
Regulating Factor	Type of Regulation	Mechanism of Regulation	Model	Ref.
CD300a/c	positive	increases IRF7 expression	Human	[89]
PDC-TREM	positive	increases the phosphorylation of PI3K, ERK1/2 and IKKα	Mouse	[90]
Ly49Q	positive	controls the intracellular trafficking of TLR9/CpG-A containing vesicular compartments	Mouse	[92]
Ly49Q	positive	increases IRF7 nuclear translocation and type I IFN gene expression	Mouse	[94]
SLAMF9	positive	supports SpiB expression	Mouse	[95]
RAGE	positive	supports transport of extracellular DNA to TLR9 (demonstrated on HEK293T cells)	mouse/human	[99,100]
**Inhibitory Receptors of Type I IFN Production**				
**Regulating Factor**	**Type of Regulation**	**Mechanism of Regulation**	**Model**	**Ref.**
BDCA2	negative	interacts with FcRγ, which activates ITAM-mediated inhibitory signals	human/mouse	[101,102,103,104]
ILT7	negative	interacts with FcRγ (FcεRIγ), which activates ITAM-mediated inhibitory signals	Human	[105]
Siglec-H	negative	interacts with DAP12, which activates ITAM-mediated inhibitory signals	Mouse	[106]
Siglec-1	negative	-	Human	[107]
LMIR8	negative	interacts with FcRγ, which activates ITAM-mediated inhibitory signals	Mouse	[108]
NKp44	negative	interacts with DAP12, which activates ITAM-mediated inhibitory signals	Human	[109]
LAIR-1	negative	through its ITIM motif recruits SHP-1 and increases its phosphatase activity (demonstrated on monocytes)	Human	[110,111]
DCIR	negative	through its ITIM motif recruits SHP-1 (demonstrated in HL-60 cells)	Mouse	[112,113]
PIR-B	negative	through SHP-1 recruitment leads to the dephosphorylation of STAT1/2	Mouse	[114]
PTPRS	negative	-	Human	[115]
PTPRF	negative	-	Mouse	[115]
EBI2	negative	through Gαi subunit of the G protein inhibits type I IFN responses	Mouse	[116]
CD28	negative	-	Mouse	[117]
TIM-3	negative	inhibits the trafficking of nucleic acids into endosomes (demonstrated on BM-DCs)	Mouse	[118]
**Receptors with Distinct Regulatory Roles: Fc Receptors**				
**Regulating Factor**	**Type of Regulation**	**Mechanism of Regulation**	**Model**	**Ref.**
FcγRIIα+ IgG containing immune complex	positive	supports TLR9 trafficking	Human	[119]
FcεRI+ IgE containing immune complex	positive	promotes the delivery of DNA to TLR9	Human	[120]
FcεRI+ free IgE	negative	triggers TNF-α, which reduces TLR9 expression	Human	[121,122]
**Interactions of Pattern Recognition Receptors**				
**Regulating Factor**	**Type of Regulation**	**Mechanism of Regulation**	**Model**	**Ref.**
TLR7-TLR9	negative	TLR7 activation inhibits TLR9-triggered IRF7 expressionand downregulates TLR9	human/mouse	[123,124]
MR-TLR9	positive	-	Mouse	[125]
TLR7-RLR	positive	TLR7 activation induces RLR expression	Human	[40]
TLR9-RLR	positive	TLR9 activation induces RLR expression	Human	[40]
NLRX1-RLR	negative	-	Human	[126]
NLRC5-RLR	negative	-	Human	[126]
TLR9-cGAS/STING	negative	cGAS/STING stimulation upregulates SOCS1 and SOCS3	Human	[127]
**Adhesion Receptors**				
**Regulating Factor**	**Type of Regulation**	**Mechanism of Regulation**	**Model**	**Ref.**
LFA-1	positive	induces TLR7 trafficking from endosomes to lysosomes	Mouse	[128]

*Abbreviations: BDCA2: blood dendritic cells antigen 2; BM-DC: bone marrow-derived dendritic cell; CD: cluster of differentiation; cGAS: cyclic GMP-AMP synthase; DAP12: DNAX activating protein of 12 kDa; DCIR: dendritic cell immunoreceptor; EBI2: Epstein-Barr virus-induced G-protein-coupled receptor 2; ERK1/2: extracellular signal-regulated kinase 1/2; FcRγ: γ subunit of the Fc receptor; FcγRIIα: Fc gamma receptor II alpha; FcεRI: Fc epsilon receptor I; FcεRIγ: γ subunit of the Fc epsilon receptor; Gαi: Gi alpha subunit; IFN: interferon; IgE: immunoglobulin E; IKKα: IκB kinase (IKK) complex α; ILT7: immunoglobulin-like transcript 7; IRF: interferon regulatory factor; ITAM: immunoreceptor tyrosine-based activation motif; ITIM: immunoreceptor tyrosine-based inhibition motif; LAIR-1: leukocyte-associated immunoglobulin-like receptor 1; LFA-1: lymphocyte function-associated antigen 1; LMIR8: leukocyte mono-immunoglobulin-like receptor 8; MR: mannose receptor; mTOR: mammalian target of rapamycin; NKp44: natural killer cell p44-related protein; NLRC5: NOD-like receptor family CARD domain containing 5; NLRX1: nucleotide-binding domain and leucine-rich repeat–containing protein X1; p70S6K: P70 S6 kinase; PDC-TREM: plasmacytoid dendritic cell—triggering receptor expressed on myeloid cells; PI3K: phosphatidylinositol 3-kinase; PIR-B: paired immunoglobulin-like receptor B; PTPRF: Protein tyrosine phosphatase receptor type F; PTPRS: Protein tyrosine phosphatase receptor type S; RAGE: receptor for advanced glycation endproducts; RLR: RIG-I-like receptor; SHP-1: Src homology 2 domain-containing protein tyrosine phosphatase 1; Siglec: sialic acid-binding immunoglobulin-type lectin; SLAMF9: signaling lymphocytic-activating molecule family 9; SOCS: suppressor of cytokine signaling; STAT: signal transducer and activator of transcription; STING: stimulator of IFN genes; TIM-3: T cell immunoglobulin and mucin domain-containing protein 3; TLR: toll-like receptor; TNF: tumor necrosis factor.*

**Table 3:** Regulation of type I IFN production by extracellular soluble factors.

Tumor-Derived Factors				
Regulating Factor	Type of Regulation	Mechanism of Regulation	Model	Ref.
Wnt5a	negative	inhibits cytoskeletal rearrangement required for cell activation	human	[159]
Wnt5a	negative	upregulates the surface expression of IDO	mouse	[160]
VIP	negative	-	human	[163,164]
TGFβ	negative	inhibits TLR9 transport to late endosomes and increases its degradation	human	[165]
TGFβ in synergy with TNFα	negative	blocks IRF7 expression and nuclear translocation	human	[167,168]
TGFβ in synergy with IL-10	negative	reduces TLR9 mRNA expression	human	[171]
IL-10	negative	-	human	[172]
PGE2 in synergy with TGFβ	negative	TGFβ induces smad, whereas PGE2 increases the levels of cAMP	human	[174]
PGE2	negative	suppresses IRF7 mRNA expression	human	[164]
HMGB1	negative	interacts with TIM-3	mouse	[118]
Gal-9	negative	through interacting with CD44 disrupts the p70S6K/mTOR signalling	human/ mouse	[178]
**Biogenic Amines and Steroid Hormones**				
**Regulating Factor**	**Type of Regulation**	**Mechanism of Regulation**	**Model**	**Ref.**
histamine	negative	acts through H2 and H4 receptors	human	[179,180]
histamine, dopamine, serotonin	negative	binds to CXCR4 and induces its internalization	human	[181]
17β-estradiol	positive	-	mouse	[182,183]
17β-estradiol	positive	increases the expression of IRF5	human	[184]
progesterone	negative	inhibits the TLR9-induced nuclear accumulation of IRF7 (mechanism demonstrated on mouse pDCs)	human/ mouse	[185]
DMPA	negative	-	mouse	[185]
MPA	negative	-	human	[186]
DHT	negative	-	human	[187]

*Abbreviations: cAMP: cyclic adenosine monophosphate; CXCR4: C-X-C motif chemokine receptor 4; DHT: dihydrotestosterone; DMPA: depot-medroxyprogesterone acetate; Gal-9: galectin-9; H2: histamine receptor 2; H4: histamine receptor 4; HMGB1: high mobility group box 1 protein; IDO: indoleamine 2,3-dioxygenase; IL: interleukin; IRF: interferon regulatory factor; MPA: medroxyprogesterone acetate; PGE2: prostaglandin E2; TGF-β: transforming growth factor beta; TIM-3: T cell immunoglobulin and mucin domain-containing protein 3; TLR: toll-like receptor; TNF: tumor necrosis factor; VIP: vasoactive intestinal peptide; Wnt5a: wingless-related integration site 5a.*

## Data Availability

Not applicable.

## Abbreviations

4E-BP	Eukaryotic translation initiation factor 4E (eIF4E)-binding protein
ACE-2	angiotensin converting enzyme 2
AIDS	Acquired Immune Deficiency Syndrome
APC	antigen presenting cell
APRIL	A proliferation-inducing ligand
APS	antiphospholipid syndrome
ARDS	acute respiratory distress syndrome
BAFF	B cell activating factor
BCR	B cell receptor
BDCA2	blood dendritic cell antigen 2
BM	bone marrow
BST2	bone marrow stromal cell antigen 2
Btk	Bruton tyrosine kinase
cAMP	cyclic adenosine monophosphate
Cbl	Casitas B cell lymphoma
CD	cluster of differentiation
CD2AP	CD2-associated adaptor protein
cDC	conventional dendritic cell
cGAS	DNA-sensing cyclic GMP-AMP synthase
CHIKV	chikungunya virus
CLE	cutaneous lupus erythematosus
COVID-19	coronavirus disease 2019
CXXC5	CXXC type zink finger protein 5
DAMP	damage-associated molecular patterns
DAP12	DNAX activation protein 12
DC	dendritic cell
DCIR	DC immunoreceptor
DENV	dengue virus
DHT	Dihydrotestosterone
DMPA	depot medroxyprogesterone acetate
DR5	death receptor 5
E2	17β-estradiol
EAE	experimental autoimmune encephalomyelitis
EBI2	Epstein-Barr virus induced receptor 2
eIF2α	eukaryotic initiation factor 2α
ER	endoplasmic reticulum
ERK	extracellular-signal-regulated kinase
ERα	estrogen receptor-α
FcR	Fc receptor
FDA	Food and Drug Administration
Flu	influenza virus
Gal-9	galectin-9
gp120	glycoprotein 120
H2	histamine receptor 2
H4	histamine receptor 4
HBsAg	hepatitis B virus surface antigen
HBV	hepatitis B virus
HCQ	Hydroxychloroquine
HCV	hepatitis C virus
HDAC3	histone deacetylase 3
HMGB1	high mobility group box 1 protein
HPC	human progenitor cells
HPV	human papilloma virus
HSV-1	herpes simplex viruses-1
IDO	indoleamine 2,3-dioxygenase
IFN	Interferon
IFNAR	interferon α receptor
IGS	interferon gene signature
IKK-α	inhibitory kappa B kinase α
IL	Interleukin
ILC2	innate lymphoid cells 2
ILT7	immunoglobulin-like transcript 7
IPC	type I interferon producing cell
IRAK	interleukin-1 receptor-associated kinase
IRF	interferon regulatory factor
ISG	interferon stimulated gene
ISGF3	interferon-stimulated gene factor 3
ISRE	interferon-stimulated response element
ITAM	immunoreceptor tyrosine-based activation motif
ITIM	immunoreceptor tyrosine-based inhibition motif
JAK1	tyrosine kinases Janus kinase 1
LAIR-1	leukocyte-associated Ig-like receptor-1
LAMP5	lysosome-associated membrane protein 5
LAP	LC3-associated phagocytosis
LCMV	lymphocytic choriomeningitis virus
LFA-1	lymphocyte functional antigen 1
LMIR8	leukocyte mono-immunoglobulin-like receptor 8
mAB	monoclonal antibodies
MAPK	mitogen-activated protein kinase
MAVS	mitochondrial antiviral signaling adapter protein
MCMV	murine cytomegalovirus
MDA5	melanoma differentiation-associated gene-5
MEK	mitogen-activated protein kinase kinase
MERS-CoV	Middle East respiratory syndrome coronavirus
MHC	major histocompatibility complex
MHV	mouse hepatitis virus
MIP-1β	macrophage inflammatory protein-1β
miRs	MicroRNAs
MR	mannose receptor
mTOR	mammalian target of rapamycin
mtROS	mitochondrial reactive oxygen species
Mx GTPAse	myxovirus resistance guanosine triphosphatase
MxA	myxovirus resistance protein 1
NCOR2	nuclear receptor co-repressor 2
NFATC3	nuclear factor of activated T cells C 3
NF-κB	Nuclear factor κ B
NK	natural killer
NLR	nucleotide-binding domain leucine-rich repeat containing receptor
OAS	Oligo A synthetase
Oligo A	2′,5′-oligoadenylate
Opn	Osteopontin
PACSIN1	Protein kinase C and casein kinase substrate in neurons 1
PAMP	pathogen-associated molecular patterns
PBMC	peripheral blood mononuclear cells
PD-1	programmed cell death 1
pDC	plasmacytoid dendritic cell
PDC-TREM	plasmacytoid dendritic cell triggering receptor expressed on myeloid cell
PGE2	prostaglandin E 2
PI3K	phosphatidylinositol 3-kinase
PIR-B	paired immunoglobulin-like receptor B
PKR	protein kinase
PLCγ2	phospholipase C-gamma 2
PLSCR1	phospholipid scramblase 1
polyU	polyuridylic acid
PRR	pattern recognition receptors
PTEN	phosphatase and tensin homolog
PTPRF	Protein tyrosine phosphatase receptor type F
PTPRS	Protein tyrosine phosphatase receptor type S
RA	Rheumatoid arthritis
RAGE	receptor for advanced glycation endproducts
RIG-I	retinoic acid-inducible gene I
RLR	RIG-I-like receptor
RNase L	ribonuclease L
ROS	reactive oxygen species
RSV	respiratory syncytial virus
RUNX2	Runt-related transcription factor 2
S1P	sphingosine-1-phosphate
SARS-CoV-2	severe acute respiratory syndrome coronavirus 2
SCARB2	Scavenger receptor class B, member 2
SH2	Src homology region 2
SHIP1	SH-2 domain-containing inositol-5-phosphatase 1
SHP-1	SH-2 domain-containing phosphatase 1
Siglec-H	sialic acid binding immunoglobulin type lectins H
SLAMF	signaling lymphocyte activation molecule family
SLE	Systemic lupus erythematosus
Slp65	Src homology 2 domain-containing leukocyte protein of 65 kDa
SOCS	suppressor of cytokine signaling
SphK1	Sphingosine kinase 1
SSc	systemic sclerosis
STAT	signal transducer and activator of transcription
STING	stimulator of interferon gene
Syk	spleen tyrosine kinase
TAB2	TGFβ activated kinase 1 binding Protein 2
TBK1	Tank-binding kinase 1
TGFβ	transforming growth factor-β
Th	T helper
TIM-3	T cell immunoglobulin and mucin domain-containing protein 3
TLR	Toll-like receptor
TNF	tumor necrosis factor
TRAF	TNF Receptor Associated Factor
TRAIL	tumor necrosis factor-related apoptosis-inducing ligand
TRIM	tripartite motif
TSC1	tuberous sclerosis complex 1
TYK2	tyrosine kinase 2
VEGFR2	vascular endothelial growth factor receptor 2
VIP	vasoactive intestinal peptide
VSV	Vesicular stomatitis virus
Wnt5a	wingless-related integration site 5a
